# M-TUBE enables large-volume bacterial gene delivery using a high-throughput microfluidic electroporation platform

**DOI:** 10.1371/journal.pbio.3001727

**Published:** 2022-09-06

**Authors:** Po-Hsun Huang, Sijie Chen, Anthony L. Shiver, Rebecca Neal Culver, Kerwyn Casey Huang, Cullen R. Buie

**Affiliations:** 1 Department of Mechanical Engineering, Massachusetts Institute of Technology, Cambridge, Massachusetts, United States of America; 2 Department of Bioengineering, Stanford University, Stanford, California, United States of America; 3 Department of Genetics, Stanford University School of Medicine, Stanford, California, United States of America; 4 Department of Microbiology and Immunology, Stanford University School of Medicine, Stanford, California, United States of America; 5 Chan Zuckerberg Biohub, San Francisco, California, United States of America; Brigham and Women’s Hospital, UNITED STATES

## Abstract

Conventional cuvette-based and microfluidics-based electroporation approaches for bacterial gene delivery have distinct advantages, but they are typically limited to relatively small sample volumes, reducing their utility for applications requiring high throughput such as the generation of mutant libraries. Here, we present a scalable, large-scale bacterial gene delivery approach enabled by a disposable, user-friendly microfluidic electroporation device requiring minimal device fabrication and straightforward operation. We demonstrate that the proposed device can outperform conventional cuvettes in a range of situations, including across *Escherichia coli* strains with a range of electroporation efficiencies, and we use its large-volume bacterial electroporation capability to generate a library of transposon mutants in the anaerobic gut commensal *Bifidobacterium longum*.

## Abstract 2

Conventional electroporation approaches for bacterial gene delivery have distinct advantages, but they are typically limited to relatively small sample volumes. This study presents a simple yet powerful microfluidic electroporation technology that enables large-scale bacterial gene delivery in a scalable manner.

## Introduction

One of the key steps in bacterial genetic engineering is the delivery of DNA into cells, which can be realized by mechanical, chemical, or electrical methods [1–3]. Among these methods, electroporation has been the gold standard because it is not cell-type-specific [2], can deliver molecules of various sizes [4], and can exhibit relatively high efficiency under optimized conditions [2,5]. For optimal electric field conditions, genetic material enters cells through reversible pores formed in the cell membrane [6,7]. Electroporation is typically performed using cuvettes in an operator-dependent manner that is limited to small batches of volume 1 mL or less. Even with high efficiency, creation of a comprehensive mutant library with hundreds of thousands of mutants [8–10] for functional-genomics studies can require electroporation of large volumes (tens of milliliters) of saturated bacterial culture, which corresponds to hundreds of cuvette-based electroporation reactions. Performing serial electroporation with manual pipetting is a labor-intensive, time-consuming, and costly process. Moreover, cuvette-based electroporation suffers from issues such as residual volume and joule heating [11,12], which affect electroporation efficiency, cell viability, and overall yield.

Performing electroporation in a microfluidic format [11–14] can remove the need for manual pipetting and improve heat dissipation [11,14], thereby increasing electroporation efficiency and cell viability. However, most microfluidic devices involve complicated fabrication processes using polydimethylsiloxane (PDMS) [15–19], which is an obstacle to widespread adoption, particularly within the microbiology community that would most benefit. Microfluidics-based electroporation devices are also typically limited by the sample volume they can handle. These devices are commonly used for mammalian cells [18,20], with just a few examples of applications to bacteria [19,21]. Several commercial products [22–26] have demonstrated the potential for scaling up electroporation to throughput of up to approximately 100 mL at 8 mL/min [26], but most have been applied only to mammalian cells and still rely on batch-wise operation [22–26]. Moreover, existing commercial systems require sophisticated electroporation chambers that limit the volume that they can process. Thus, the capabilities of these systems for large-volume bacterial electroporation are yet unproved.

The ideal genetic transformation system would allow for a wide range of sample volumes to accommodate different applications, especially involving the creation of mutant libraries given the low electroporation efficiency of many understudied yet health-relevant bacterial species [10,27,28]. A scalable, high-volume electroporation device should be easily assembled by a microbiologist without sophisticated fabrication, compatible with commercially available and common laboratory equipment, and able to process relevant sample volumes in minutes to minimize biological variability. To this end, here we introduce a simple yet powerful Microfluidic TUbing-based Bacterial Electroporation (M-TUBE) device that enables flexible electroporation of large-volume bacterial samples. M-TUBE facilitates scalable, continuous flow, large-volume bacterial electroporation without the need for micro/nanofabrication, PDMS casting, or 3D printing of microfluidic channels and electrodes.

## Results

### Assembly and characterization of the M-TUBE device

**Fig 1:**
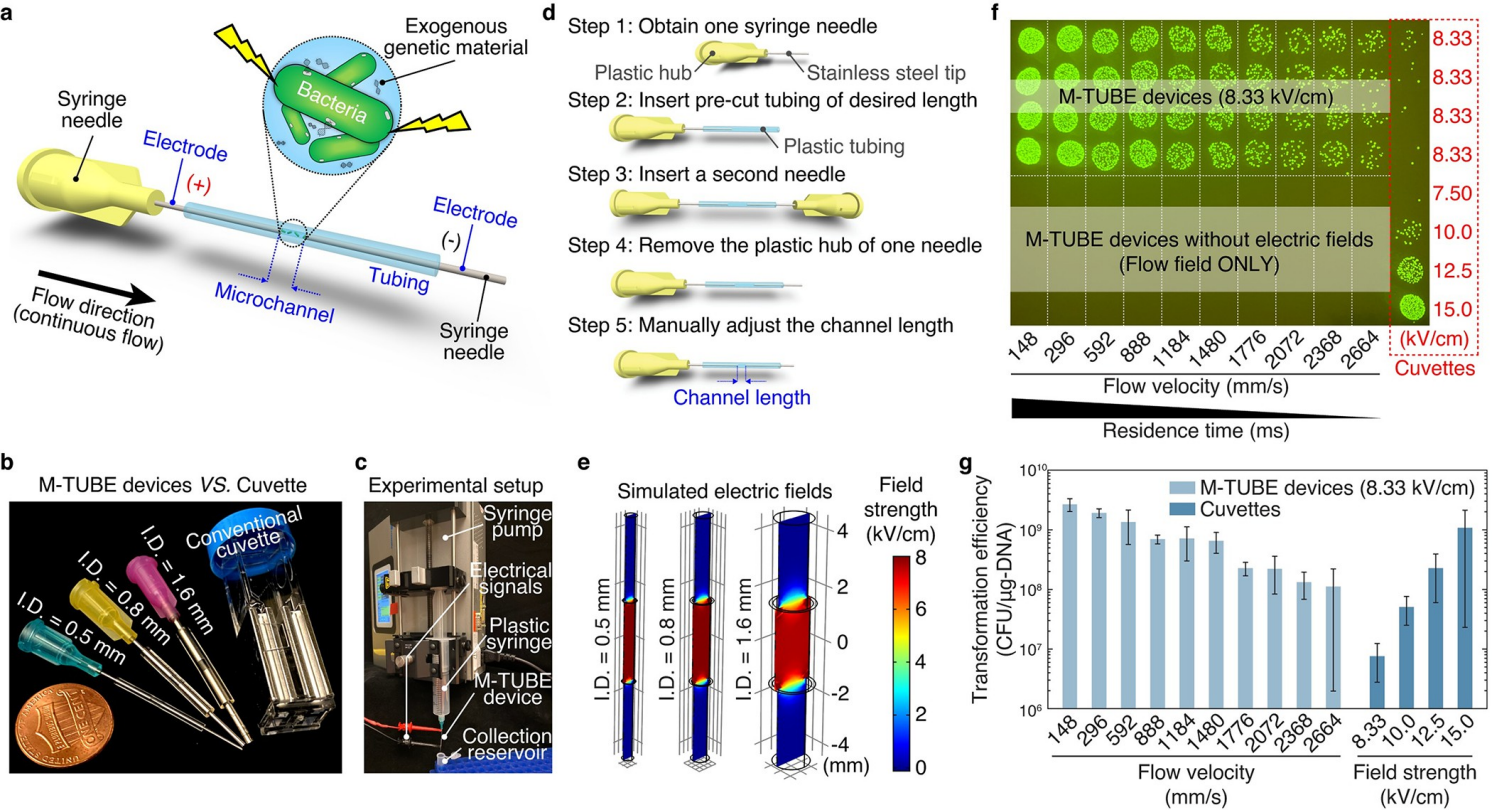
M-TUBE is a fabrication-free, microfluidics tubing-based bacterial electroporation device that is simple to assemble and exhibits higher electroporation efficiency than cuvettes. (a) Schematic of the M-TUBE device. The device is composed of 2 syringe needles and 1 piece of plastic tubing of predefined length. The 2 syringe needles and plastic tubing serve as the 2 electrodes and microchannel, respectively. When the 2 electrodes are connected to an external power supply (or electrical signal generator), an electric field is established within the microchannel, where bacterial electroporation can take place. (b) M-TUBE devices with 3 ID are all similar in size to a conventional cuvette. (c) Photograph of the experiment setup when using the M-TUBE device. Since the M-TUBE device is made from standard, commercially available syringe needles and plastic tubing, it can be readily attached to syringe pumps for automated sample delivery, removing the need for manually pipetting samples. (d) Detailed breakdown of the protocol for M-TUBE assembly. One device can be completely assembled in 90–120 s. The total cost of parts is currently less than $0.22 and this price could be lowered if parts are bought in bulk. (e) Simulations of the electric field established in M-TUBE devices using COMSOL Multiphysics predict similar field strengths irrespective of ID. (f) Spot-dilution assay to quantify viability on selective plates when *E*. *coli* NEB10β cells were flowed through the device with a plasmid encoding ampicillin resistance and GFP (S4 Table) in the presence or absence of an electric field. Transformation was dependent on the electric field. For M-TUBE devices, a voltage of ±2.50 kV (AC field) was applied, which results in an electric field of 8.33 kV/cm. The same batch of cells was used to conduct cuvette-based electroporation as a comparison. (g) Comparison of transformation efficiency (CFUs per μg of DNA) corresponding to the plates in (f). The electroporation efficiency of M-TUBE decreased as the fluid velocity was increased, as expected due to the shorter duration of exposure to the electric field. Regardless of the fluid velocity, the efficiency of M-TUBE was at least 1 order of magnitude higher than that of cuvettes with the same field strength (8.33 kV/cm). Data represent the average (*n* ≥ 3) and error bars represent 1 standard deviation. The data underlying Fig 1E and 1G can be found in S1 and S2 Data files, respectively. AC, alternating current; CFU, colony-forming unit; GFP, green fluorescent protein; ID, inner diameter; M-TUBE, microfluidic tubing-based bacterial electroporation.

The M-TUBE device consists of 2 syringe needles and 1 plastic tube of a defined length (**Fig 1A**). The plastic tubing serves as the microfluidic channel, and the syringe needles serve as the 2 electrodes, which, when connected to an external high-voltage power supply (Methods), establish an electric field across the tubing microchannel. Upon establishing an electric field in the channel, bacterial cells flowing through the channel can be electrotransformed and uptake surrounding genetic materials. The syringe needles and plastic tubing used to assemble M-TUBE are commercially and readily available at low cost (<$0.21 per device), and the overall size of an M-TUBE device is similar to that of a conventional cuvette (**Fig 1B**). Because syringe needles of standard common formats can be used, M-TUBE can be attached to any commercially available syringe with complementary connectors and can be conveniently interfaced with any syringe pump for sample delivery (**Fig 1C**).

The M-TUBE device can be easily assembled in 5 steps (**Fig 1D**). In brief, device assembly is accomplished by inserting 1 syringe needle into the plastic tubing cut to a particular length (Methods), and a second syringe needle is inserted into the other end of the tubing. Once both needles are inserted, the length of the channel is manually adjusted to a predefined value (Methods) by modifying the gap between the facing ends of the 2 syringe needles. Assembling a single M-TUBE device requires only 90 to 120 s (Methods and **S1 Video**), far more convenient than typical fabrication processes for microfluidic devices (which usually require several days).

Simulations of the electric field established in the tubing microchannel of M-TUBE (**Fig 1E**) indicate that the electric field strength is unaffected by the size of the microchannel (i.e., the tubing inner diameter (ID)), assuming that the applied voltage (e.g., 2.50 kV) and distance between the 2 electrodes (gap or microchannel length) are held constant. This characteristic enables M-TUBE devices to cover a wider range of sample flow rates without having to adjust the applied voltage to maintain the same field strength. The gap of M-TUBE devices can be easily adjusted without additional assembly, unlike devices that rely on microfabrication, CNC machining, or 3D printing [29], providing a simple method for adjusting the electric field strength of a device. Another beneficial feature is that the residence time within M-TUBEs can be adjusted to control cell exposure to the electric field. Since M-TUBE electroporates bacterial cells in a continuous flow manner, the residence time is dictated by the fluid velocity (or flow rate), such that residence time decreases with an increase in fluid velocity if the gap is fixed (**S1 Table**). These 2 features, gap length and flow rate, offer users more flexibility in tuning important electroporation parameters such as the electric field strength and the residence time, respectively, which are not always readily tunable in conventional electroporators.

### Optimization of bacterial electroporation with M-TUBE

To establish the utility of M-TUBE, optimize its design, and showcase its ability to electrotransform bacterial cells, we used a strain of *Escherichia coli* (NEB10β) with high transformation efficiency. The M-TUBE devices employed for most experiments conducted in this study were comprised of a 500-μm diameter tube and 3-mm gap and were supplied with a voltage of ±2.50 kV or 5.00 kV_PP_ (peak-to-peak AC signal, square wave), which leads to a field strength of 8.33 kV/cm within the microchannel. Cuvettes with 2-mm gaps were used to perform electroporation at different voltages as a control. We first confirmed that the flow field (or flow shear stress) along the tube does not by itself lead to genetic transformation. In the absence of an electric field, simply flowing cells through M-TUBE at fluid velocities ranging from 148 mm/s (1.8 mL/min) to 2,664 mm/s (32.6 mL/min) did not result in any transformation events (**Fig 1F**, bottom). By contrast, once a sufficient electric field was established within M-TUBE, colonies were obtained across the entire range of flow rates tested (**Fig 1F**, top), with transformation efficiencies ranging from 10^8^ to 10^10^ colony-forming units (CFUs)/μg of DNA (**Fig 1G**). A reduction in electroporation efficiency was observed as the fluid velocity was increased. This trend was expected because the residence time decreases as the flow rate increases; hence, cells are exposed to the electric field for a shorter duration at higher flow rates. Despite the lower efficiency at higher flow rates, the overall efficiency obtained using the M-TUBE device was at least 1 order of magnitude higher than that obtained using cuvettes with the same field strength (8.33 kV/cm). We also note that, compared to cuvettes (typically used at 10 to 15 kV/cm), M-TUBE was able to produce a comparable efficiency using a lower electric field. Cell viability after electroporation was of similar magnitude using M-TUBE devices as with cuvettes (S1 Note and S1 Fig). The finding that M-TUBE outperforms cuvettes in terms of transformation efficiency may be due to a synergistic effect of the flow field and the electric field [30].

**Fig 2:**
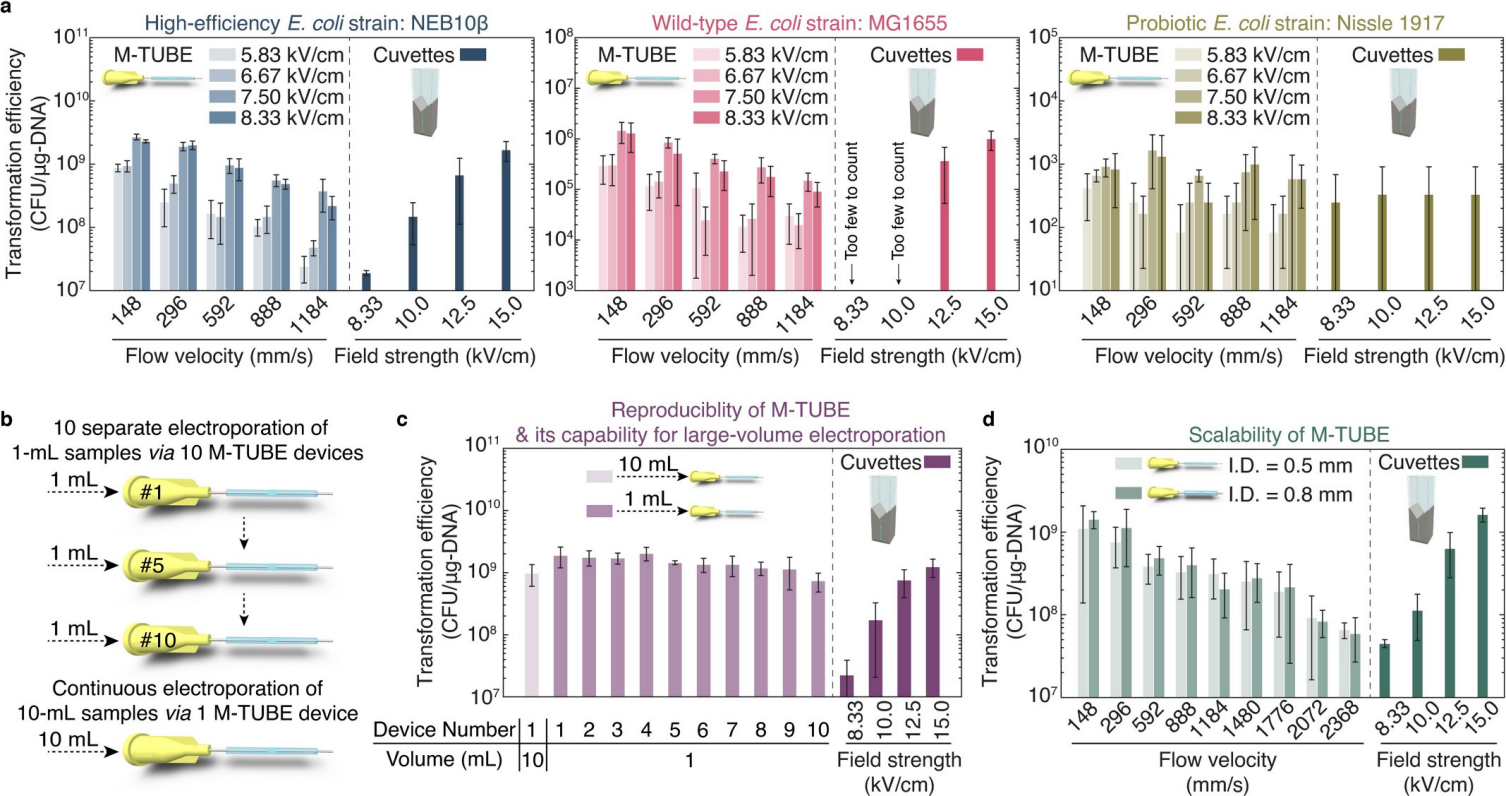
The M-TUBE device exhibits higher efficiency than cuvettes across *E*. *coli* strains, is reproducible, and maintains high efficiency across tubing sizes. (a) Comparison of M-TUBE device performance when transforming the high-efficiency strain NEB10β, the wild-type strain MG1655, and the probiotic strain Nissle 1917 across voltages and fluid velocities. M-TUBE outperformed cuvettes at an equivalent electric field strength for all strains. Data represent the average (*n* ≥ 3) and error bars represent 1 standard deviation. (b) Schematic of the experiment comparing 10 separate 1 mL electroporations and 1 continuous electroporation of a 10-mL sample. (c) Transformation efficiency for the experiments in (b) demonstrates that sample volume can be increased without compromising efficiency. Data represent the average (*n* ≥ 3) and error bars represent 1 standard deviation. The same batch of cells was used to conduct cuvette-based electroporation as a comparison. (d) Transformation efficiency was similar across 0.5-mm and 0.8-mm diameter M-TUBE devices. For M-TUBE devices, a voltage of ±2.50 kV (AC field) was applied, which results in an electric field of 8.33 kV/cm. Data represent the average (*n* ≥ 3) and error bars represent 1 standard deviation. The data underlying this figure can be found in S2 Data. AC, alternating current; M-TUBE, microfluidic tubing-based bacterial electroporation.

Given the strong dependence of transformation efficiency on field strength in cuvette-based electroporation, we next evaluated how M-TUBE performs across field strengths. Compared to cuvette-based electroporation at 8.33 kV/cm, regardless of the supplied field strength, M-TUBE exhibited higher transformation efficiencies across the range of flow rates tested (**Fig 2A**, left). This finding indicates that M-TUBE can either achieve the same efficiency with lower field strengths or higher efficiency with the same field strength. Moreover, electroporation efficiencies with M-TUBE had a smaller standard deviation than those obtained with cuvette-based electroporation. Thus, M-TUBE provides several benefits compared with cuvettes in addition to its high-volume capability.

Most M-TUBE electroporation experiments in this study were carried out using an electric field generated with alternating current (AC) rather than direct current (DC). With DC fields, M-TUBE exhibited higher electroporation efficiency than cuvettes using the same field strength or comparable efficiency using a lower field strength, although efficiency and reproducibility with DC fields were overall lower than with AC fields (S3 Fig). To determine whether M-TUBE transformation efficiency depends on AC field frequency, we conducted electroporation experiments across 5 fluid velocities in the range 148 to 1,184 mm/s with a distinct frequency (50, 100, 200, 300, 400 Hz) for each fluid velocity so that cells flowing through the microchannel were exposed to only a single pulse (S2 Fig). For a comparison, electroporation was also carried out at a common frequency (400 Hz) for all fluid velocities tested. Electroporation efficiency was largely independent of AC field frequency (S2 Fig). This result contrasted with a previous study that observed frequency dependence [31], potentially due to differences in channel geometry. Regardless, our findings highlight the flexibility of M-TUBE.

### M-TUBE exhibits comparable or better efficiency compared with cuvettes across *E*. *coli* strains

Motivated by the successful transformation of *E*. *coli* NEB10β, M-TUBE was then tested on the wild-type strain *E*. *coli* MG1655, which typically has lower transformation efficiency than NEB10β. The results show that M-TUBE maintained higher efficiency than cuvettes for MG1655 (**Fig 2**, middle). With a field strength of 8.33 kV/cm, M-TUBE yielded efficiencies at least 2 orders of magnitude higher than cuvettes; even though cuvettes were supplied with a field strength of 10 kV/cm, the number of successfully transformed colonies was too low to reliably enumerate. To further test M-TUBE performance on *E*. *coli* strains, we used M-TUBE to electroporate the probiotic strain Nissle 1917 [27,28]. While both M-TUBE and cuvettes exhibited much lower electroporation efficiencies for Nissle 1917 compared with MG1655, M-TUBE was comparably efficient to cuvettes and showed slightly better reproducibility (**Fig 2A**, right). Moreover, the ability of M-TUBE to process arbitrarily large sample volumes in a continuous fashion means that a desired number of transformed cells of a low-efficiency strain such as Nissle can be obtained with M-TUBE simply by processing a sufficiently large volume. Conversely, using cuvettes for the same goal would be expensive and technically challenging. Overall, M-TUBE showed robust performance across *E*. *coli* strains with a wide range of electroporation efficiencies, with performance and reproducibility higher than or comparable to cuvette-based electroporation.

### Assembly has negligible effect on reproducibility of M-TUBE

Since M-TUBE is hand assembled, small fluctuations in the microchannel length are inevitable across independently assembled M-TUBE devices (even assembled by the same user). Given that the field strength is defined as the ratio of the applied voltage to the microchannel length, we sought to evaluate if the field strength differs significantly across identical but separately assembled M-TUBE devices, thereby causing variation in electroporation performance for NEB10β cells (**Fig 2B**, top). We concurrently carried out electroporation of a large-volume sample (10 mL) to demonstrate the capacity of M-TUBE for high-volume electroporation (**Fig 2B**, bottom), from which we were able to determine if there is a substantial difference in transformation efficiency between multiple small-volume electroporation experiments and continuous flow large-volume electroporation. The variation across 10 M-TUBE devices was nonsignificant and negligible, and each of the tested devices outperformed cuvettes regardless of the field strength (**Fig 2C**), confirming that assembly has negligible impact on the reproducibility of the M-TUBE.

Furthermore, M-TUBE was able to electroporate the entire 10-mL sample at a flow rate of 3.6 mL/min with efficiency higher than or comparable to cuvettes (**Fig 2C**), and the transformation efficiency for 10 mL of continuous electroporation was not significantly different from that of 10 separate 1-mL experiments. Continuous electroporation of 10 mL is equivalent to 100 individual 0.1-mL cuvette-based electroporations, for which the configuration of M-TUBE that we tested would shorten the entire electroporation time by 2 to 3 orders of magnitude (depending on the flow rate). Put in other terms, M-TUBE can process 2 to 3 orders of magnitude more volume of sample in a given period of time compared with cuvettes (**S2 Table**). In terms of cost, M-TUBE is at least 10-fold cheaper than cuvettes (**S3 Table**). Moreover, using M-TUBE for large-volume bacterial electroporation can also circumvent the need for manual pipetting by flowing the electroporated sample directly into recovery medium (**S2 Video**), thereby decreasing total processing time and potentially improving cell viability and transformation efficiency. Taken together, these features make M-TUBE an ideal candidate for large-volume bacterial electroporation.

### M-TUBE throughput can be scaled up without compromising efficiency

Our next goal was to evaluate the ability to scale up the M-TUBE to process even larger volume samples. To this end, the performance of the M-TUBE device with 3 different IDs was compared (500, 800, and 1,600 μm, with the size of syringe needles altered accordingly) (**Figs 2D and S4**). As long as the gap and the fluid velocity were held fixed, M-TUBE devices with different diameters maintained a high electroporation efficiency for NEB10β cells and outperformed cuvettes. With the same fluid velocity, an M-TUBE device with larger diameter would enable processing larger volumes: with a diameter of 1,600 μm, an average fluid velocity of 592 mm/s allows for electroporation of approximately 70 mL/min, several orders of magnitude more than what is possible with cuvettes. These results again demonstrate the capabilities of M-TUBE for large-volume bacterial electroporation and confirm that M-TUBE can be readily scaled up without compromising efficiency simply by changing the tubing and syringe needles sizes while maintaining fluid velocity.

### Numerical evaluation of Joule heating in M-TUBE devices

Compared to electroporation of mammalian cells (tens of microns in size), which typically requires electric field strengths <2 kV/cm, successful electroporation of bacterial cells (approximately 1 μm in size) requires field strengths of 10 to 25 kV/cm. The use of large electric fields introduces the risk of increased Joule heating, which could compromise cell viability. To estimate the magnitude of Joule heating in M-TUBE devices, we conducted numerical modeling of the temperature distribution inside an M-TUBE microchannel under various conditions (Methods). For a fluid velocity of 148 mm/s (**S5A Fig**), which corresponds to a residence time of approximately 20 ms, simulations predicted a localized temperature increase between approximately 2°C and approximately 15°C for an applied electric field of 8.33 kV/cm, dependent on the transient location of cells while flowing through the microchannel.

While simulations predicted a maximum temperature increase of up to 15°C, cells would be exposed to these high temperatures for only a short period of time (<20 ms even for the slowest fluid velocities), and simulations predicted that flowing cells at the faster fluid velocity of 592 mm/s, which corresponds to a residence time of approximately 5 ms, would improve heat dissipation by providing better cooling and thereby lower the maximum temperature increase and even out the spatial distribution of temperatures (**S5B Fig**). Application of lower electric field strengths would also be beneficial for reducing the Joule heating effect (**S6 Fig**). Moreover, we confirmed numerically that the temperature increase (Δ*T*) is independent of the initial temperature of the cell sample (**S7 Fig**). These results suggest that cell samples should be suspended in relatively cold electroporation buffer so that the final temperature inside the channel is below approximately 40°C, beyond which cell viability could be compromised (although many species may be able to survive extremely short periods of heating). Taken together, these simulations indicate that when M-TUBE devices are used to electroporate cells using high-magnitude electric fields, the optimal conditions are higher fluid velocities, buffers with lower conductivities, and cell suspensions in cold buffers.

### Generation of a transposon mutant library in an anaerobic gut commensal with M-TUBE

**Fig 3:**
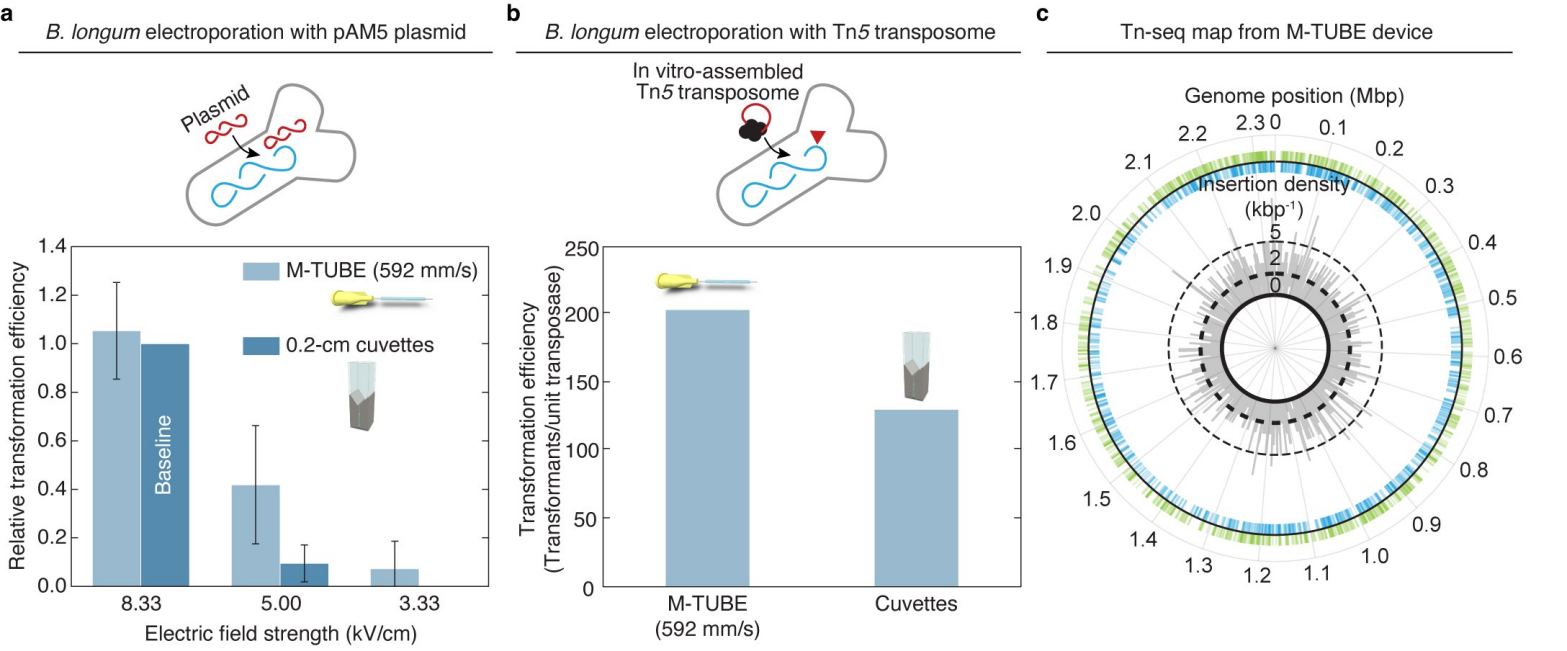
M-TUBE efficiently transforms anaerobic bacteria and enables transposon insertion mutagenesis. (a) Comparison of M-TUBE performance during electrotransformation of *B*. *longum* NCIMB8809 with the plasmid pAM5 at various electric field strengths. For M-TUBE devices, voltages of ±2.50, ±1.50, and ±1.00 kV (AC field) were applied to produce electric fields of 8.33, 5.00, and 3.33 kV/cm, respectively. A fluid velocity of 592 mm/s was used for the M-TUBE device because approximately 5 ms residence time with an M-TUBE ID of 0.5 mm is similar to the time constant observed in cuvette electroporation (5.2–5.6 ms). Data represent the average (*n* ≥ 3) and error bars represent 1 standard deviation. (b) Comparison of M-TUBE performance during electrotransformation of *B*. *longum* NCIMB8809 with Tn*5* transposome. For the M-TUBE device, a field strength of 8.33 kV/cm and fluid velocity of 592 mm/s were used, motivated by the results in (a). (c) The transposon insertions recovered from Tn*5* transposome electroporation are spread approximately uniformly across the *B*. *longum* NCIMB8809 genome. The locations of individual mapped insertions are recorded on the outer circle. Green ticks on the outside indicate insertions in the positive (+) orientation, blue ticks on the inside indicate insertions in the negative (−) orientation. The insertion density (kbp^−1^) (both positive and negative orientation) is plotted in 1-kbp bins on the inner circle. Transposon insertions are distributed throughout the genome in both the positive and negative orientations, indicating that *B*. *longum* NCIMB8809 can be transformed by Tn*5* transposomes using M-TUBE without major insertional bias. The data underlying this figure can be found in S2 Data. AC, alternating current; ID, inner diameter; M-TUBE, microfluidic tubing-based bacterial electroporation.

As a demonstration of the utility of M-TUBE in other organisms, we sought to use the system to generate a set of transposon insertion mutants in a human gut commensal. Many of these organisms are obligate anaerobes and hence require more complex handling during growth, washing, and electroporation. We assembled the M-TUBE electroporation platform inside an anaerobic chamber and ran an experiment to generate a small-scale transposon insertion pool in *Bifidobacterium longum* subsp. *longum* NCIMB8809. *B*. *longum* species are used as probiotics and are actively investigated for their health-promoting effects [32]. To identify optimal electroporation conditions for maximizing transposome delivery, we first electroporated *B*. *longum* NCIMB8809 cells with the pAM5 plasmid (**Fig 3A** and **S4 Table**). As with *E*. *coli*, M-TUBE plasmid transformation efficiency was comparable to or higher than that of cuvettes for *B*. *longum* (**Fig 3A**). With the optimal electroporation conditions, *B*. *longum* cells were successfully transformed with *in vitro*-assembled EZ-Tn*5* transposomes, demonstrating its utility both in an anaerobic chamber and for high-throughput transposon mutagenesis (**Fig 3B and 3C**). Like plasmids, M-TUBE transposome electroporation efficiency was comparable to or higher than that of cuvettes. Transposon sequencing of the transformants revealed >2,000 unique transposition events spread across the genome (**Fig 3C** and **S5 Table**). Given these encouraging results, we expect that a scaled-up transformation protocol will produce a transposon pool of sufficient diversity for future chemical-genomic investigation using barcode sequencing [8,33,34]. Furthermore, we expect M-TUBE should have wide applicability for generation of libraries of thousands of transposon mutants, even in bacterial species with complex growth requirements.

## Conclusions

Taken together, the disposable, fabrication-free M-TUBE device can process large volumes of bacterial cells with dramatically reduced processing time and effort, without compromising transformation efficiency and cell viability. Due to the simplicity of its fabrication and the wide availability of its components, M-TUBE presents an electroporation strategy that can be immediately implemented in the microbiology community. The flexibility that M-TUBE offers in tuning electroporation conditions such as field strength and residence time make the device a powerful tool for working with hard-to-transform strains. Given the relatively high transformation efficiency compared with cuvettes and its ability to deal with both small and large volumes, M-TUBE has the potential to be a viable alternative to cuvettes and an indispensable tool for applications requiring large volumes such as the creation of mutant libraries.

## Methods

### Materials

Syringe needles of various gauges (16, 20, or 23) with blunt tips were purchased from CML Supply LLC. Plastic tubing of various diameters were purchased from Cole-Parmer: 0.5-mm ID (PB-0641901), 0.8-mm ID (EW-07407-70), and 1.6-mm ID (EW-07407-71). Plastic syringes of various volumes with Luer-Lok tips were purchased from Thomas Scientific: 30 mL (BD302832), 20 mL (BD302830), and 10 mL (BD302995). Luria broth (LB) (BD244620) and dehydrated agar (BD214010) were purchased from VWR. MRS broth (BD288130) and reinforced clostridial medium (RCM) (CM0149B) were purchased from Fisher Scientific. Carbenicillin disodium salt (C3416), tetracycline (T7660), L-cysteine (C7352), α-lactose monohydrate (L2643), and sucrose (S7903) were purchased from Millipore Sigma. Oligonucleotides (S4 Table) were purchased from Integrated DNA Technologies.

### Modeling electric field strength and temperature distribution in an M-TUBE microchannel

To simulate the electric field within M-TUBE devices as a function of plastic tubing diameter and the temperature distribution under various electroporation conditions, we built a numerical model in COMSOL Multiphysics v. 6.0 (Burlington, Massachusetts). The model is based on the multiphysics module of electromagnetic heating, which couples the physics of electric currents, laminar flow, and heat transfer in solids and fluids. To reduce the computational complexity of the model, we used a simplified channel geometry 500 μm in diameter and 3 mm in length that only includes the tips of the 2 needle electrodes and the microchannel formed between the electrodes. Equations, boundary conditions, assumptions, and numerical techniques used to compute electric fields, flow fields, and temperatures are similar to previous studies [19,35,36]. To conservatively model the temperature distribution inside an M-TUBE microchannel, we assumed that 10% (v/v) glycerol contributed to the electrical conductivity with 0.01 S/m [37–39].

### Protocol for preparation of an M-TUBE device

An M-TUBE device is assembled from 2 syringe needles and 1 piece of plastic tubing with a predefined length (**Fig 1D** and **S1 Video**). Here, we describe the details of assembly of an M-TUBE device with a microchannel length of 3 mm and a tubing ID of 0.5 mm. First, we cut plastic tubing (50 feet per roll) into 20-mm segments on a cutting mat with metric dimensions. Second, we take 2 syringe needles of 23 gauge with a tip length of 0.5 in, which has an outer diameter of 0.63 mm that ensures tight fitting between the tubing inner surface and the outer surface of the syringe needle. Next, we insert one of the syringe needles into the tubing and repeatedly rotate back and forth the tubing and/or syringe needle until the tip of the syringe needle is close to the middle of the tubing, and there is also a small portion of the needle for electrical connection that is not inserted into the tubing. We then insert the other syringe needle and rotate back and forth the tubing/syringe needle or the second syringe needle until a gap (i.e., the microchannel length) of 2 to 4 mm between the tips of the 2 syringe needles is established. The gap size can be checked by placing the entire assembly close to a tape measure. After assembling the 3 components, we remove the plastic hub from either of the syringe needles. Upon removal of the plastic hub, the gap size should then be carefully rechecked with a tape measure, and slight adjustments can be made to establish a gap of 3 mm by gently twisting either needle inward or outward. After this final adjustment, the M-TUBE device is completely assembled.

As discussed above, assembly of 1 M-TUBE device requires only 60 to 90 s; hence, we typically prepare 50 M-TUBE devices at a time, in approximately 1 h. The M-TUBE devices are placed in a Petri dish, which is sterilized in a biosafety cabinet with UV irradiation overnight. After UV sterilization, M-TUBE devices are stored in a −20°C freezer or refrigerator until just before conducting electroporation experiments, a step similar to the prechilling of electroporation cuvettes.

To prepare M-TUBE devices with other tubing sizes, all steps remain unchanged, but it is necessary to ensure that the plastic tubing is assembled with syringe needles that have complementary outer diameters in their tips.

### The external high-voltage power supply system

The external high-voltage power supply (S8 **Fig**) consists of a function generator (Agilent Technologies, 33220A), a high-voltage amplifier (Trek, 623B), and an oscilloscope (Agilent Technologies, DSO-X 2022A). The function generator supplies preprogrammed electric signals (AC or DC, sine or square waves, frequency, voltage, etc.) to the high-voltage amplifier, which amplifies the signals by 1,000-fold. The oscilloscope monitors the amplified signals to ensure the correct output. The function generator provides non-amplified signals to the amplifier through a BNC cable, and the amplifier outputs amplified signals through a pair of high-voltage cables, which were customized with alligator clips or test clips and connected to the 2 electrodes of an M-TUBE device. On/off switching of the high-voltage signals was primarily controlled by engaging and disengaging a trigger button on the function generator. The function generator, amplifier, and oscilloscope used in this study are standard electronic equipment that can be accessed in many research laboratories/facilities or readily acquired.

### Culturing and preparation of *E*. *coli* strains

Three *E*. *coli* strains, including NEB10β (New England Biolabs), K-12 MG1655 (Coli Genetic Stock Center, Yale University), and Nissle 1917 (Mutaflor, Canada), were employed in this study to test the M-TUBE device. The strains, unless otherwise specified, were cultured, harvested, and made electrocompetent using the same conditions. In brief, glycerol stocks were inoculated into two 14-mL cultures tubes containing 6 mL of LB medium and incubated at 37°C and 250 rpm. The next morning, 5 mL from each overnight culture were inoculated into 245 mL of LB and grown at 37°C and 200 rpm to an OD_600_ of 0.5 to 0.7. Note that each set of *E*. *coli* experiments involved 15 to 20 mL of electrocompetent cells at OD_600_ = 10, which required two 250-mL cultures. Each 250 mL culture was divided equally into six 50-mL centrifuge tubes and spun down at 4°C and 3,500 rpm for 10 min using an Allegra 64R centrifuge (Beckman Coulter). The supernatant was discarded and 6 mL of ice-cold 10% glycerol were used to wash and combine the 6 cell pellets into 1 suspension. Each 6-mL cell suspension was equally divided into four 2.0-mL microcentrifuge tubes. The 8 microcentrifuge tubes generated from the two 250-mL cultures were centrifuged at 4°C and 8,000 rpm for 5 min, the supernatants were discarded, and 1 mL of ice-cold 10% glycerol was used to wash and resuspend the pellet in each of the 8 tubes. These washing steps were repeated twice more. Next, all cell pellets were combined into a concentrated suspension using 8 mL of ice-cold 10% glycerol, and the cell concentration (typically OD_600_ = 20 to 30) was measured using a UV spectrophotometer (UV-1800, Shimadzu). Depending on the measured concentration, a final sample with OD_600_ = 10 was prepared by adding an appropriate volume of ice-cold 10% glycerol. This sample was placed on ice prior to electroporation. DNA plasmids (Parts Registry K176011) [19] encoding ampicillin resistance and green fluorescent protein (GFP) were added to this sample at a final concentration of 0.1 ng/μL for NEB10β and MG1655 cultures; for Nissle 1917, a final concentration of 1 ng/μL was employed so that the number of CFUs was above the limit of detection. For electroporation, the sample was loaded into a 30-mL plastic syringe.

### *B*. *longum* culturing and preparation for M-TUBE electroporation with plasmid DNA

A 5-mL *B*. *longum* culture was maintained in an anaerobic chamber (Coy) via daily dilution into fresh medium to prepare for electroporation. Briefly, 1 mL of a *B*. *longum* culture was inoculated into 9 mL of MRS medium in a culture tube, and 5 additional serially diluted (at 1:10 ratio) cultures were prepared; these 6 cultures were incubated at 37°C overnight. The next morning, the optical density of each culture was measured using a spectrometer, and the culture with OD_600_ = 3 to 4 was used for subsequent outgrowth. The selected culture was diluted to OD_600_ = 0.54 in 60 to 70 mL and grown to OD_600_ = 1.5 to 2, after which cells were harvested and made electrocompetent following the same steps described above for *E*. *coli*. The 60 to 70 mL were then divided equally into two 50-mL centrifuge tubes and spun down outside the anaerobic chamber at 4°C and 3,500 rpm for 10 min using an Allegra 64R ultracentrifuge (Beckman Coulter). Next, the two 50-mL centrifuge tubes were returned to the anaerobic chamber, the supernatant was discarded, and 5 mL of ice-cold 10% glycerol were used to wash and combine the 2 cell pellets into 1 suspension. The 5-mL cell suspension was divided equally into four 2-mL microcentrifuge tubes. The 4 tubes were centrifuged inside the chamber at room temperature and 10,000 rpm for 2 min using an Eppendorf 5418 microcentrifuge, the supernatants were discarded, and 1 mL of ice-cold 10% glycerol was used to wash and resuspend the pellet in each of the 4 tubes. These washing steps were repeated 2 more times. Next, all pellets were combined into a concentrated suspension using 5 mL of ice-cold 10% glycerol. Depending on the concentration, the final sample at OD_600_ = 10 was prepared by adding the appropriate volume of ice-cold 10% glycerol and then placed on ice prior to electroporation. The pAM5 plasmid encoding tetracycline resistance was added to the sample at a final concentration of 2 ng/μL. The mixture of the plasmid DNA with the cells was loaded into a 10-mL plastic syringe for electroporation.

### Transposon mutagenesis of *B*. *longum* NCIMB8809

Previous transformation protocols [40–42] were combined with minor modifications to prepare electrocompetent cells of *B*. *longum* NCIMB8809. Briefly, a glycerol stock of *B*. *longum* NCIMB8809 was recovered for 24 h in 5 mL of MRS broth (MRS media, Difco) at 37°C and passaged overnight (16 h) in 10 mL of MRS in a 10-fold dilution series. The next morning, the incubator temperature was raised to 40°C and 1 of the overnight cultures in the dilution series was used inoculate 50 mL of MRS (MRS media, HIMEDIA) in a 250-mL Erlenmeyer flask at an initial OD_600_ (optical density at λ = 600 nm) of 0.18, as measured by a 96-well plate reader (Epoch2, BioTek) in a 96-well flat bottom microplate (Grenier Bio-One, Cat. #655161) with 200 μL of culture per well. In the dilution series, the overnight culture with the lowest optical density that still provided enough cells to proceed was used to inoculate the next culture. The 50 mL of culture in HIMEDIA-brand MRS were grown to an OD_600_ of 1.0 and used to inoculate MRS broth reconstituted from individual components, modified with 1% lactose as the sole carbon source and an additional 133 mM NaCl, at an initial OD_600_ of 0.18. This culture was harvested at an OD_600_ of 0.5, pelleted, washed 3 times with 15% glycerol, and resuspended at an OD_600_ of 6.7 in 15% (v/v) glycerol. To harvest the cells, the culture was moved to a prereduced 50 mL conical tube (Fisher Scientific, Cat. #06-443-19) on ice, brought out of the anaerobic chamber, centrifuged for 10 min at 3,428*g* (Centrifuge 5920R, Eppendorf), and transferred back into the anaerobic chamber. After cells were harvested, the incubator temperature was lowered back down to 37°C. Subsequent washes were performed at a volume of 5 mL in 5-mL Eppendorf tubes (Cat. #0030122321, Eppendorf) and pelleted with a compatible microcentrifuge (MC-24 Touch, Benchmark Scientific) that had been brought into the chamber, using 2-min 10,000*g* centrifugation steps. Transposomes were assembled *in vitro* by mixing an erythromycin resistance cassette with commercially available EZ-Tn*5* transposase according to manufacturer’s instructions. Transposomes were mixed with competent cells at a concentration of 2U transposase/mL competent cells and electroporated using the M-TUBE device (see below). Electroporated cells were recovered for 2 h at 37°C, concentrated 10-fold through centrifugation and resuspension in MRS, and plated on RCM-agar plates with 5 μg/mL erythromycin. Colonies were harvested for sequencing after approximately 36 h of growth at 37°C.

### Electroporation of *E*. *coli* strains using M-TUBE

The final sample of cells mixed with plasmid DNA was loaded into a plastic syringe, which was mounted on a syringe pump (Legato 210P, KD Scientific) that could be operated horizontally or vertically. To prevent bending of the plastic tubing of the M-TUBE device and to enable convenient collection of the electroporated sample directly into tubes, we typically operate the syringe pump as shown in **Fig 1C**. After arranging the pump to operate vertically, an M-TUBE device was attached to the sample-loaded syringe via Luer-Lok connection, and the 2 syringe-needle electrodes were connected to the external high-voltage power supply system (S8 **Fig**). Upon confirming a tight connection between the M-TUBE device and the power supply, we prefilled the M-TUBE microchannel by infusing the cell sample at a relatively low flow rate (typically 250 to 500 μL/min), to prevent air bubbles and thereby arcing/sparking in M-TUBE, until we visually confirmed that the microchannel was filled with the liquid cell sample. Next, a collection tube (reservoir) was placed underneath the M-TUBE device (**Fig 1C**) so that the electroporated sample could be directly and automatically collected. We programmed the pumping parameters including target pumping volume and pumping flow rate and started flow using the syringe pump at the preset flow rate; immediately after starting flow, we started the application of electric signals to the M-TUBE device to initiate electroporation.

As a positive control, the same batch of electrocompetent cells was also electroporated at various field strengths using 0.2-cm electroporation cuvettes (VWR, 89047–208). One hundred microliters were pipetted into a prechilled electroporation cuvette. Each cuvette was pulsed with an electroporator (MicroPulser, Bio-Rad) at field strengths including 8.33 kV/cm, 10.0 kV/cm, 12.5 kV/cm, and 15 kV/cm with time constants between 5.0 to 5.5 ms. Immediately after the application of electric pulses to each cuvette, 900 μL of prewarmed (approximately 37°C) LB recovery medium were added to each cuvette, and the 100-μL electroporated cells was mixed with the 900-μL recovery medium via pipetting. We then aspirated as much electroporate sample volume as possible from the cuvette and dispensed it into designated wells on a 96-well deep plate (**S9 Fig**), along with the electroporated samples from M-TUBE for subsequent recovery at 37°C for 1 h.

### Electroporation of *B*. *longum* via M-TUBE

Most steps for *B*. *longum* were the same as for *E*. *coli* described above; the differences are described here. After prefilling an M-TUBE device with the *B*. *longum* sample, a 50-mL conical tube (reservoir) containing MRS recovery medium was placed underneath the M-TUBE device (**S10 Fig**) so that electroporated *B*. *longum* cells could be directly and automatically flowed into the recovery medium. For *B*. *longum* electroporation with M-TUBE, 1 flow rate (7.2 mL/min or 592 mm/s for the 0.5-mm M-TUBE device) was tested at 3 field strengths (3.33, 5.00, and 8.33 kV/cm).

As a positive control, the same batch of electrocompetent cells was electroporated at the same 3 field strengths using 0.2-cm electroporation cuvettes. One hundred microliters of the final cell sample were pipetted into a prechilled electroporation cuvette. Each cuvette was pulsed by the electroporator with time constants ranging between 5.4 to 5.8 ms. Immediately after the application of an electric pulse, 1,000 μL of prewarmed (approximately 37°C) LB recovery medium were added to each cuvette and mixed with the cells via pipetting. We then aspirated as much electroporated sample volume as possible from the cuvette and dispensed it into a 1.5-mL microcentrifuge tube.

### Collection, recovery, and evaluation of electroporated *E*. *coli* samples

In each set of *E*. *coli* experiments, a range of flow rates and electric field strengths were tested; for each combination of testing conditions, 1 mL of electroporated sample was collected in a microcentrifuge tube. One hundred microliters of the electroporated sample were aspirated and dispensed into each of 4 wells of a 96 deep-well plate containing LB recovery medium (**S9 Fig**). In each 96-well plate, we were able to test 20 combinations of electroporation conditions. After filling all designated wells of the 96-well plate, the plate was incubated in a shaking incubator at 37°C and 250 rpm for 1 h. After 1 h of recovery, the 96-well sample plate was placed in a designated position on a liquid handling robot (Janus BioTx Pro Plus, PerkinElmer) for automated serial dilution (**S11 Fig**): 10×, 100×, and 1,000× dilution for *E*. *coli* NEB10β; 10× and 100× dilution for *E*. *coli* K12 MG1655 or Nissle 1917. Following serial dilution, 5 μL from each well were dispensed onto LB-agar plates containing 50 μg/mL carbenicillin, and the selective plates were incubated overnight at 37°C. The next morning, each plate was photographed for CFU counting.

### Collection, recovery, and evaluation of electroporated *B*. *longum* samples

After electroporating *B*. *longum* using M-TUBE, 1 mL of cells was flowed directly into 10 mL of MRS recovery medium. *B*. *longum* samples electroporated by M-TUBE or in cuvettes were incubated at 37°C for 3 h. Following recovery, 1.1 mL from each M-TUBE or cuvette sample were aspirated and pipetted into separate 1.5-mL microcentrifuge tubes and spun down at 10,000 rpm for 2 min. The supernatants were discarded and 200 μL of MRS medium were added into each 1.5-mL tubes to resuspend the cell pellets. Next, the 200-μL suspension was plated onto RCM-agar plates with 10 μg/mL tetracycline, and the selective plates were incubated at 37°C for at least 48 h. Following the 48-h incubation, each plate was photographed for CFU counting.

### CFU quantification

Photos of selective plates for electroporation with plasmids were captured using an iPhone 11 (Apple) on a tripod with a remote shutter. The photos were imported to ImageJ (NIH) and CFU.Ai v. 1.1 for enumerating CFUs. The transformation efficiency was defined as the number of CFUs on selective plates per μg of DNA.

### Preparing a Tn-seq library for *B*. *longum* NCIMB8809

Erythromycin-resistant colonies from the Tn*5* transposome electroporation were scraped from the selective plates into 500 μL of MRS broth (MRS media, Difco) for each Petri dish. Samples from this suspension were taken, glycerol (Fisher Bioreagents, Cat. #BP229-1) was added to a final concentration of 15% (v/v), and cryostocks were stored in 11-mm crimp vials (Thermo Scientific, Cat. #C4011-11) with sealed aluminum crimp caps (Thermo Scientific, Cat. #11-03-400) at −80°C. Simultaneously, most of the suspension was stored directly at −20°C for subsequent DNA isolation. Genomic DNA (gDNA) was isolated from the colony suspension using a DNeasy Blood and Tissue Kit (QIAGEN, Cat. #69506) according to the manufacturer’s protocol for Gram-positive organisms.

Isolated gDNA was first sheared in a Covaris S220 Sonicator with microTUBE AFA fiber preslit snap-cap tubes (Covaris, Cat. #520045) according to the manufacturer’s instructions for 300-bp fragments. A KAPA HyperPrep Kit (Roche, 07962312001) with custom oligos was then used to prepare the library. Briefly, sonicated gDNA was subjected to a dual bead-based size selection using AMPure XP magnetic beads (Beckman Coulter, Cat. #A63881) according to the manufacturer’s instructions for 300-bp sized fragments. An end-repair and A-tailing reaction was performed followed by an adaptor ligation by following the KAPA HyperPrep protocol and using a custom adaptor (**S4 Table**). After a 1-sided bead cleanup, the entire sample of adaptor-ligated gDNA fragments was used as input for a PCR reaction that simultaneously amplified transposon-gDNA junctions and added Illumina TruSeq adaptors. An Ultra II Q5 PCR mix (New England Biolabs, Cat. #E7649A) was used for all PCR reaction components except the template DNA and custom primers (**S4 Table**). The PCR reaction involved an initial denaturation step of 98°C for 2 min, followed by 25 cycles of 3 steps: 98°C for 30 s, 65°C for 20 s, and 72°C for 30 s. After a final extension at 72°C for 10 min, the sample was cleaned up using a NucleoSpin Gel and PCR Clean-up kit (Machery-Nagel, Cat. #740609.250). The Tn-seq library was run on a MiSeq (Illumina, Cat. #SY-410-1003), with a 150-cycle MiSeq Reagent Kit V3 (MS-102-3001), 150-bp read 1 length, and no indexing reads.

## Supporting information

S1 Data Data underlying Fig 1E. (XLSX)

S2 Data Data underlying Figs 1G, 2, 3, and S1–S4. (XLSX)

S1 Note Comparison of cell viability between M-TUBE and conventional cuvettes. (DOCX)

S1 Table Residence time (the duration that cells were exposed to electric fields in M-TUBE devices) as a function of fluid velocities (or flow rates). (DOCX)

S2 Table Comparison of processing times between conventional cuvettes and M-TUBE devices. (DOCX)

S3 Table Comparison of costs for assembly of an M-TUBE device versus a cuvette per unit processing volume. (DOCX)

S4 Table Strains, plasmids, and oligos used in this study. (DOCX)

S5 Table List of transposition events. (TXT)

S1 Fig Comparison of transformation efficiency and cell viability between M-TUBE devices and conventional cuvettes. For M-TUBE devices, a voltage of ±2.50 kV (AC field) was applied, which results in an electric field of 8.33 kV/cm. Data represent the average (*n* ≥ 3) and error bars represent 1 standard deviation. The data underlying this figure can be found in S2 Data. AC, alternating current; M-TUBE, microfluidic tubing-based bacterial electroporation.
(TIF)

S2 Fig Dependence of M-TUBE transformation efficiency on the frequency of the applied AC field. With M-TUBE devices, electroporation efficiency was largely independent of the applied AC field frequency. For M-TUBE devices, a voltage of ±2.50 kV (AC field) was applied, which results in an electric field of 8.33 kV/cm. Data represent the average (*n* ≥ 3) and error bars represent 1 standard deviation. The data underlying this figure can be found in S2 Data. AC, alternating current; M-TUBE, microfluidic tubing-based bacterial electroporation.
(TIF)

S3 Fig M-TUBE device performance is higher using AC fields compared with DC fields. Using DC fields, M-TUBE devices achieved higher transformation efficiency than cuvettes using the same field strength or comparable efficiency using a lower field strength. Overall, electroporation efficiency and reproducibility were lower using DC fields compared with AC fields. For M-TUBE devices, a voltage of ±2.50 kV (AC field) or 2.50 kV (DC field with a duty cycle of 95%) was applied, which results in an electric field of 8.33 kV/cm. Data represent the average (*n* ≥ 3) and error bars represent 1 standard deviation. The data underlying this figure can be found in S2 Data. AC, alternating current; DC, direct current; M-TUBE, microfluidic tubing-based bacterial electroporation.
(TIF)

S4 Fig Transformation efficiency is maintained across M-TUBE devices with different diameters. To further evaluate the scalability of M-TUBE, M-TUBE devices made using plastic tubing with 0.5-mm, 0.8-mm, and 1.6-mm inner diameters and compared to conventional cuvettes. A voltage of ±2.50 kV (AC field) was applied to each M-TUBE device, resulting in an electric field of 8.33 kV/cm. The same batch of cells was used to conduct electroporation with 0.2-mm cuvettes and various voltages as a comparison. Data represent the average (*n* ≥ 3) and error bars represent 1 standard deviation. The data underlying this figure can be found in S2 Data. AC, alternating current; M-TUBE, microfluidic tubing-based bacterial electroporation.
(TIF)

S5 Fig Simulated temperature distribution in an M-TUBE microchannel as a function of fluid velocity. Cell samples were introduced into the microchannel at a fluid velocity of (a) 148 mm/s or (b) 592 mm/s. Simulations predicted higher and more uneven temperature increases for lower fluid velocity. The M-TUBE geometry used for simulations was 500 μm in diameter and 3 mm in length, and a voltage of 2.50 kV was applied, which leads to an electric field of 8.33 kV/cm. The initial temperature of the cell sample was 20°C. M-TUBE, microfluidic tubing-based bacterial electroporation.
(TIF)

S6 Fig Simulated temperature distribution in an M-TUBE microchannel as a function of voltage. Voltages of (a) 2.25 kV (7.50 kV/cm) or (b) 2.00 kV (6.67 kV/cm) were applied. The M-TUBE geometry used for simulations was 500 μm in diameter and 3 mm in length. Cell samples were flowed through the microchannel at a fluid velocity of 592 mm/s for both simulations. The initial temperature of the cell sample was 20°C. M-TUBE, microfluidic tubing-based bacterial electroporation.
(TIF)

S7 Fig Simulated temperature distribution in an M-TUBE microchannel as a function of initial temperature. The cell sample was initialized with a temperature of (a) 20°C or (b) 4°C before flowing through the channel. The M-TUBE geometry used for simulations was 500 μm in diameter and 3 mm in length, and a voltage of 2.50 kV was applied, which leads to an electric field of 8.33 kV/cm. Cell samples flowed through the microchannel at a fluid velocity of 148 mm/s for both simulations. M-TUBE, microfluidic tubing-based bacterial electroporation.
(TIF)

S8 Fig Image of the high-voltage power supply system. The system is composed of a function generator that allows for waveform programming, a high-voltage amplifier applied to the signal from the function generator, and an oscilloscope that allows for real-time monitoring of the amplified signal.
(TIF)

S9 Fig Schematic of the arrangement of electroporation conditions tested in a 96-well deep-well plate. One milliliter of electroporated cells was collected for each combination of electroporation conditions tested. One hundred microliters were dispensed from each 1-mL sample into each of 4 designated wells containing 900 μL of LB recovery medium. For cuvette experiments, all of the volume aspirated from each cuvette was dispensed into a well. LB, Luria broth.
(TIF)

S10 Fig Photograph of M-TUBE set up in an anaerobic chamber. The M-TUBE device can be easily and conveniently set up in an anaerobic chamber. The photograph also shows that placing a collection tube (reservoir) directly underneath the fluid as it exits the M-TUBE device would enable the direct and automated transfer of electroporated cells into recovery medium. M-TUBE, microfluidic tubing-based bacterial electroporation.
(TIF)

S11 Fig Workflow employing a commercial liquid-handling robot for automated liquid transfer and serial dilution. After 1 h of recovery, the 96-well deep plate that contains electroporated samples was mounted on a liquid-handling robot. By leveraging the capabilities of the robot, we used the M-TUBE device to test a wide range of electroporation conditions, each with at least 3–4 technical replicates, while removing the need for extensive manual pipetting for sample transfer, sample dilution, and sample plating. Strain shown is *E*. *coli* NEB10β. M-TUBE, microfluidic tubing-based bacterial electroporation.
(TIF)

S1 Video The detailed procedure for assembling an M-TUBE device, demonstrating that the entire process to assemble an M-TUBE device requires only 60–90 s. (MP4)

S2 Video Electroporated cells can be directly and automatically flowed into the recovery medium, removing the need for extensive manual pipetting. (MP4)

## Abbreviations

AC: alternating current
CFU: colony-forming unit
DC: direct current
gDNA: genomic DNA
GFP: green fluorescent protein
ID: inner diameter
LB: Luria broth
M-TUBE: microfluidic tubing-based bacterial electroporation
PDMS: polydimethylsiloxane
RCM: reinforced clostridial medium
