# Supplementary material for: M-TUBE enables large-volume bacterial gene delivery using a high-throughput microfluidic electroporation platform
Source: PLoS Biol. 2022 Sep 6;20(9):e3001727. doi: 10.1371/journal.pbio.3001727 (PMC9481174; doi:10.1371/journal.pbio.3001727)
Supplement: S1 Note — (DOCX) [file pbio.3001727.s003.docx]

**Supplementary Note 1**

**M-TUBE enables large-volume bacterial gene delivery using a high-throughput microfluidic electroporation platform**

Po-Hsun Huang^1^, Sijie Chen^1,†^, Anthony L. Shiver^2,†^, Rebecca Neal Culver^3^,

Kerwyn Casey Huang^2,4,5^, Cullen R. Buie^1,*^

**Affiliations:**

^1^Department of Mechanical Engineering, Massachusetts Institute of Technology, Cambridge, MA 02139

^2^Department of Bioengineering, Stanford University, Stanford, CA 94305

^3^Department of Genetics, Stanford University School of Medicine, Stanford, CA 94305

^4^Department of Microbiology and Immunology, Stanford University School of Medicine, Stanford, CA 94305

^5^Chan Zuckerberg Biohub, San Francisco, CA 94158

^†^: These authors contributed equally to this manuscript.

**Comparison of cell viability between M-TUBE and conventional cuvettes**

One factor that impacts transformation efficiency is the viability of cells after electroporation. We conducted three sets of experiments to measure cell viability (survival rate) after electroporation using M-TUBE devices or conventional cuvettes. The survival rate is defined as the ratio of the number of viable cells from the electroporated sample compared with a non-electroporated sample. M-TUBE devices, compared to cuvette-based electroporation at 8.33 kV/cm, exhibited higher transformation efficiencies across the range of flow rates tested, despite a survival rate of ~50% for M-TUBE electroporation versus ~100% with cuvettes (Fig. S1). Cuvette-based electroporation at 15.0 kV/cm showed similar efficiency and survival rate as M-TUBE devices at lower field strengths (Fig. S1). Taken together, while cell viability from electroporation with M-TUBE devices may be slightly lower than with cuvettes, transformation efficiency is not impacted.
