# Supplementary material for: M-TUBE enables large-volume bacterial gene delivery using a high-throughput microfluidic electroporation platform
Source: PLoS Biol. 2022 Sep 6;20(9):e3001727. doi: 10.1371/journal.pbio.3001727 (PMC9481174; doi:10.1371/journal.pbio.3001727)
Supplement: S1 Table — (DOCX) [file pbio.3001727.s004.docx]

**Supplementary Table 1**

**Table S1:** **Residence time (the duration that cells were exposed to electric fields in M-TUBE devices) as a function of fluid velocities (or flow rates).**

| **M-TUBE device with tubing inner diameter (ID) = 0.5 mm** | | | | | | | | | | |
| --- | --- | --- | --- | --- | --- | --- | --- | --- | --- | --- |
| **Flow velocity**  **(mm/s)** | ~148 | ~296 | ~592 | ~888 | ~1184 | ~1480 | ~1776 | ~2072 | ~2368 | ~2664 |
| **Flow rate**  **(mL/min)** | 1.8 | 3.6 | 7.2 | 10.8 | 14.4 | 18.0 | 21.6 | 25.2 | 28.8 | 32.4 |
| **Residence time**  **(ms)** | **~20.27** | **~10.13** | **~5.07** | **~3.08** | **~2.53** | **~2.03** | **~1.69** | **~1.45** | **~1.27** | **~1.13** |
| **M-TUBE device with tubing ID = 0.8 mm** | | | | | | | | | | |
| **Flow velocity**  **(mm/s)** | ~148 | ~296 | ~592 | ~888 | ~1184 | ~1480 | ~1776 | ~2072 | ~2368 | ~2664 |
| **Flow rate**  **(mL/min)** | 4.4 | 8.8 | 17.6 | 26.4 | 35.2 | 44.0 | 52.8 | 61.6 | 70.4 | 79.2 |
| **Residence time**  **(ms)** | **~20.27** | **~10.13** | **~5.07** | **~3.08** | **~2.53** | **~2.03** | **~1.69** | **~1.45** | **~1.27** | **~1.13** |
| **M-TUBE device with tubing ID = 1.6 mm** | | | | | | | | | | |
| **Flow velocity**  **(mm/s)** | ~148 | ~296 | ~592 | ~888 | ~1184 | ~1480 | ~1776 | ~2072 | ~2368 | ~2664 |
| **Flow rate**  **(mL/min)** | 17.6 | 35.2 | 70.4 | 105.6 | 140.8 | 176.0 | 211.2 | 246.4 | 281.6 | 316.8 |
| **Residence time**  **(ms)** | **~20.27** | **~10.13** | **~5.07** | **~3.08** | **~2.53** | **~2.03** | **~1.69** | **~1.45** | **~1.27** | **~1.13** |
