# Supplementary material for: M-TUBE enables large-volume bacterial gene delivery using a high-throughput microfluidic electroporation platform
Source: PLoS Biol. 2022 Sep 6;20(9):e3001727. doi: 10.1371/journal.pbio.3001727 (PMC9481174; doi:10.1371/journal.pbio.3001727)
Supplement: S2 Table — (DOCX) [file pbio.3001727.s005.docx]

**Supplementary Table 2**

**Table S2:** **Comparison of processing times between conventional cuvettes and M-TUBE devices.** The processing times of cuvettes and M-TUBE devices both scale linearly with sample volume. Across all flow velocities and sample volumes, the M-TUBE device exhibits substantially lower processing time than cuvettes.

| **0.2-cm cuvette (1-1.5 minutes for 100 μL)** | | | | | | | | |
| --- | --- | --- | --- | --- | --- | --- | --- | --- |
| **Processing volume** | | **1 mL** | **5 mL** | **10 mL** | **50 mL** | **100 mL** | **500 mL** | **1,000 mL** |
| Processing time | | 10-15 min | 50-75 min | 1.6-2.5 h | 8-12 h | 16-25 h | 83-125 h | 165-250 h |
|  | | | | | | | | |
| **M-TUBE device (processing time is dependent of flow rates used)** | | | | | | | | |
| **Processing volume** | | **1 mL** | **5 mL** | **10 mL** | **50 mL** | **100 mL** | **500 mL** | **1,000 mL** |
| M-TUBE  (Inner diameter (ID) = **0.5 mm**)  Processing time (min) | 148 mm/s  (1.8 mL/min) | 0.56 | 2.78 | 5.56 | 27.78 | 55.56 | 277.78 | 555.56 |
|  | 296 mm/s  (3.6 mL/min) | 0.28 | 1.39 | 2.78 | 13.89 | 27.78 | 138.89 | 277.78 |
|  | 592 mm/s  (7.2 mL/min) | 0.14 | 0.69 | 1.39 | 6.94 | 13.89 | 69.44 | 138.89 |
|  | 888 mm/s  (10.8 mL/min) | 0.09 | 0.46 | 0.93 | 4.63 | 9.26 | 46.30 | 92.59 |
|  | 1184 mm/s  (14.4 mL/min) | 0.07 | 0.35 | 0.69 | 3.47 | 6.94 | 34.72 | 69.44 |
| M-TUBE  (ID = **0.8 mm**)  Processing time (min) | 148 mm/s  (4.4 mL/min) | 0.23 | 1.14 | 2.27 | 11.36 | 22.73 | 113.64 | 227.27 |
|  | 296 mm/s  (8.8 mL/min) | 0.11 | 0.57 | 1.14 | 5.68 | 11.36 | 56.82 | 113.64 |
|  | 592 mm/s  (17.6 mL/min) | 0.06 | 0.28 | 0.57 | 2.84 | 5.68 | 28.41 | 56.82 |
|  | 888 mm/s  (26.4 mL/min) | 0.04 | 0.19 | 0.38 | 1.89 | 3.79 | 18.94 | 37.88 |
|  | 1184 mm/s  (35.2 mL/min) | 0.03 | 0.14 | 0.28 | 1.42 | 2.84 | 14.20 | 28.41 |
| M-TUBE  (ID = **1.6 mm**)  Processing time (min) | 148 mm/s  (17.6 mL/min) | 0.06 | 0.28 | 0.57 | 2.84 | 5.68 | 28.41 | 56.82 |
|  | 296 mm/s  (35.2 mL/min) | 0.03 | 0.14 | 0.28 | 1.42 | 2.84 | 14.20 | 28.41 |
|  | 592 mm/s  (70.4 mL/min) | 0.01 | 0.07 | 0.14 | 0.71 | 1.42 | 7.10 | 14.20 |
|  | 888 mm/s  (105.6 mL/min) | 0.01 | 0.05 | 0.09 | 0.47 | 0.95 | 4.73 | 9.47 |
|  | 1184 mm/s  (140.8 mL/min) | 0.01 | 0.04 | 0.07 | 0.36 | 0.71 | 3.55 | 7.10 |
