# Supplementary material for: M-TUBE enables large-volume bacterial gene delivery using a high-throughput microfluidic electroporation platform
Source: PLoS Biol. 2022 Sep 6;20(9):e3001727. doi: 10.1371/journal.pbio.3001727 (PMC9481174; doi:10.1371/journal.pbio.3001727)
Supplement: S3 Table — (DOCX) [file pbio.3001727.s006.docx]

**Supplementary Table 3**

**Table S3:** **Comparison of** **costs for assembly of one M-TUBE device versus cuvettes per unit processing volume.**

| **Parts cost for M-TUBE devices and conventional cuvettes** | | | | | | |
| --- | --- | --- | --- | --- | --- | --- |
| **Parts for M-TUBE** | | **Quantity** | | **Bulk Price (USD)** | **Note** |  |
| Syringe needle | | 1000 pieces | | <$101.9 | (<$0.10 per piece) |  |
| Plastic tubing | | 3048 cm | | <$58.9 | (<$0.02 per cm) |  |
|  | |  | |  | **(One M-TUBE device costs <$0.22)** |  |
| **Parts for cuvettes** | | **Quantity** | | **Bulk Price (USD)** | **Note** |  |
| 0.2-cm cuvette from VWR | | 50 pieces | | $111.38 | ($2.23 per cuvette) |  |
| 0.2-cm cuvette from BIO-RAD | | 50 pieces | | $117.75 | ($2.36 per cuvette) |  |
|  | | | | | | |
| **Cost to electroporate a unit volume sample (parts only)** | | | | | | |
| **Processing volume** | **0.2-cm cuvette from VWR** | | **0.2-cm cuvette from BIO-RAD** | | **M-TUBE device** |  |
| 1 mL | $22.30 | | $23.60 | | <$0.22 |  |
| 5 mL | $111.38 | | $117.75 | | <$0.22 |  |
| 10 mL | $222.76 | | $235.50 | | <$0.22 |  |
| 50 mL | $1,113.80 | | $1,177.50 | | <$0.22 |  |
| 100 mL | $2,227.60 | | $2,355.00 | | <$0.22 |  |
| 500 mL | $11,138.00 | | $11,775.00 | | <$0.22 |  |
| 1000 mL | $22,276.00 | | $23,550.00 | | <$0.22 |  |
