# Supplementary material for: M-TUBE enables large-volume bacterial gene delivery using a high-throughput microfluidic electroporation platform
Source: PLoS Biol. 2022 Sep 6;20(9):e3001727. doi: 10.1371/journal.pbio.3001727 (PMC9481174; doi:10.1371/journal.pbio.3001727)
Supplement: S4 Table — (DOCX) [file pbio.3001727.s007.docx]

**Supplementary Table 4**

**Table S4: Strains, plasmids, and oligos used in this study.**

| **Strain** | | **Source** | |
| --- | --- | --- | --- |
| *E. coli* NEB10β | | New England Biolabs | |
| *E. coli* K-12 MG1655 | | Yale Coli Genetics Stock Center | |
| *E. coli* Nissle 1917 | | Mutaflor | |
| *Bifidobacterium longum* subsp. *longum* NCIMB8809 | | Gift from Douwe van Sinderen, University College Cork, Ireland | |
| **Plasmid** | **Reference** | **Source** | |
| pCon1.00 (J23100)->RBS+GFP+T | <http://parts.igem.org/Part:BBa_K176011> | iGEM | |
| pAM5 | [1] | Gift from Douwe van Sinderen, University College Cork, Ireland | |
| **Oligo** | **Sequence­** | **Description** | **Source** |
| Erm-For | /5Phos/CTGTCTCTTATACACATCTATTTATGTTACAGTAATATTGACTTCGACACC | Forward primer for generating randomly barcoded erm^R^ transposon | This study |
| Erm-Rev | /5Phos/CTGTCTCTTATACACATCT GTCGACCTGCAGCGTACG NNNNNNNNNNNNNNNNNNNN AGAGACCTCGTGGACATC TTACACATTATTCCGGTGATAGGGC | Reverse primer for generating randomly barcoded erm^R^ transposon | This study |
| Adaptor-A | /5Phos/GATCGGAAGAGCACACGTCTGAACTCCAGTCA | First half of adaptor for Tn-seq library preparation | Reference [2] |
| Adaptor-B | ACGCTCTTCCGATC*T | Second half of adaptor for Tn-seq library preparation. ‘*’ mark is shorthand for a phosphorothioate bond. | Reference [2] |
| Tnseq-For | ATGATACGGCGACCACCGAGATCTACACTCTTTCC CTACACGACGCTCTTCCGATCT NNNNNN GATGTCCACGAGGTCT | Forward primer for Tn-seq library amplification, Tn*5* binding, introduces P5 | Reference [2] |
| Tnseq-Rev | CAAGCAGAAGACGGCATACGAGAT ATTGGC GTGACTGGAGTTCAGACGTGTGCTCTTCCGATCT | Reverse primer for Tn-seq library amplification, adaptor binding, introduces P7 | Reference [2] |

**References**

1. Alvarez-Martin P, O'Connell-Motherway M, van Sinderen D, Mayo B. Functional analysis of the pBC1 replicon from Bifidobacterium catenulatum L48. Appl Microbiol Biotechnol. 2007;76(6):1395-402. Epub 2007/08/21. doi: 10.1007/s00253-007-1115-5. PubMed PMID: 17704917.

2. Wetmore KM, Price MN, Waters RJ, Lamson JS, He J, Hoover CA, et al. Rapid quantification of mutant fitness in diverse bacteria by sequencing randomly bar-coded transposons. mBio. 2015;6(3):e00306-15. Epub 2015/05/15. doi: 10.1128/mBio.00306-15. PubMed PMID: 25968644; PubMed Central PMCID: PMCPMC4436071.
