# Supplementary figures and images for: M-TUBE enables large-volume bacterial gene delivery using a high-throughput microfluidic electroporation platform
Source: PLoS Biol. 2022 Sep 6;20(9):e3001727. doi: 10.1371/journal.pbio.3001727 (PMC9481174; doi:10.1371/journal.pbio.3001727)

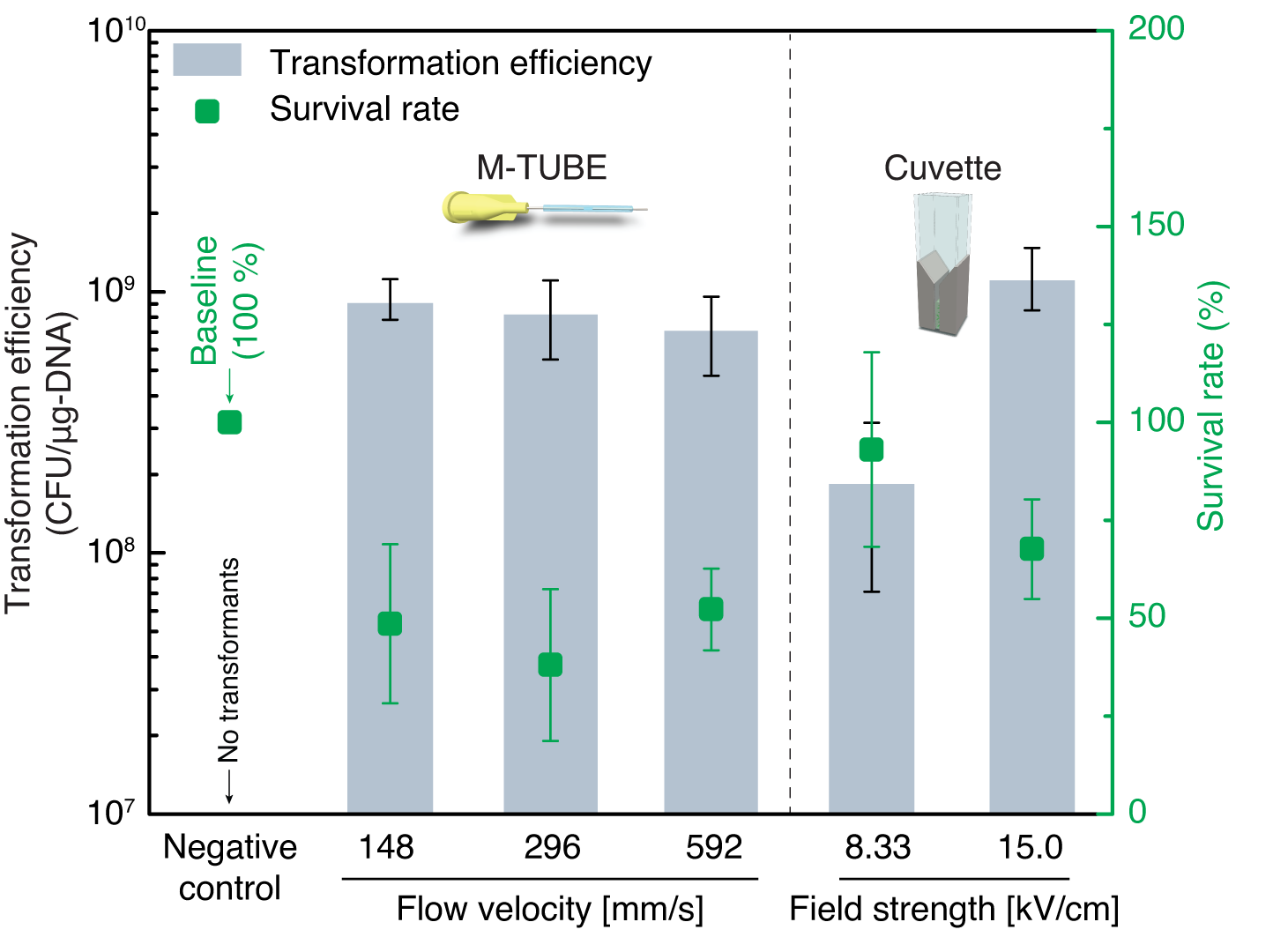

Supplement: S1 Fig — For M-TUBE devices, a voltage of ±2.50 kV (AC field) was applied, which results in an electric field of 8.33 kV/cm. Data represent the average (n ≥ 3) and error bars represent 1 standard deviation. The data underlying this figure can be found in S2 Data. AC, alternating current; M-TUBE, microfluidic tubing-based bacterial electroporation. (TIF) [file pbio.3001727.s009.tif]

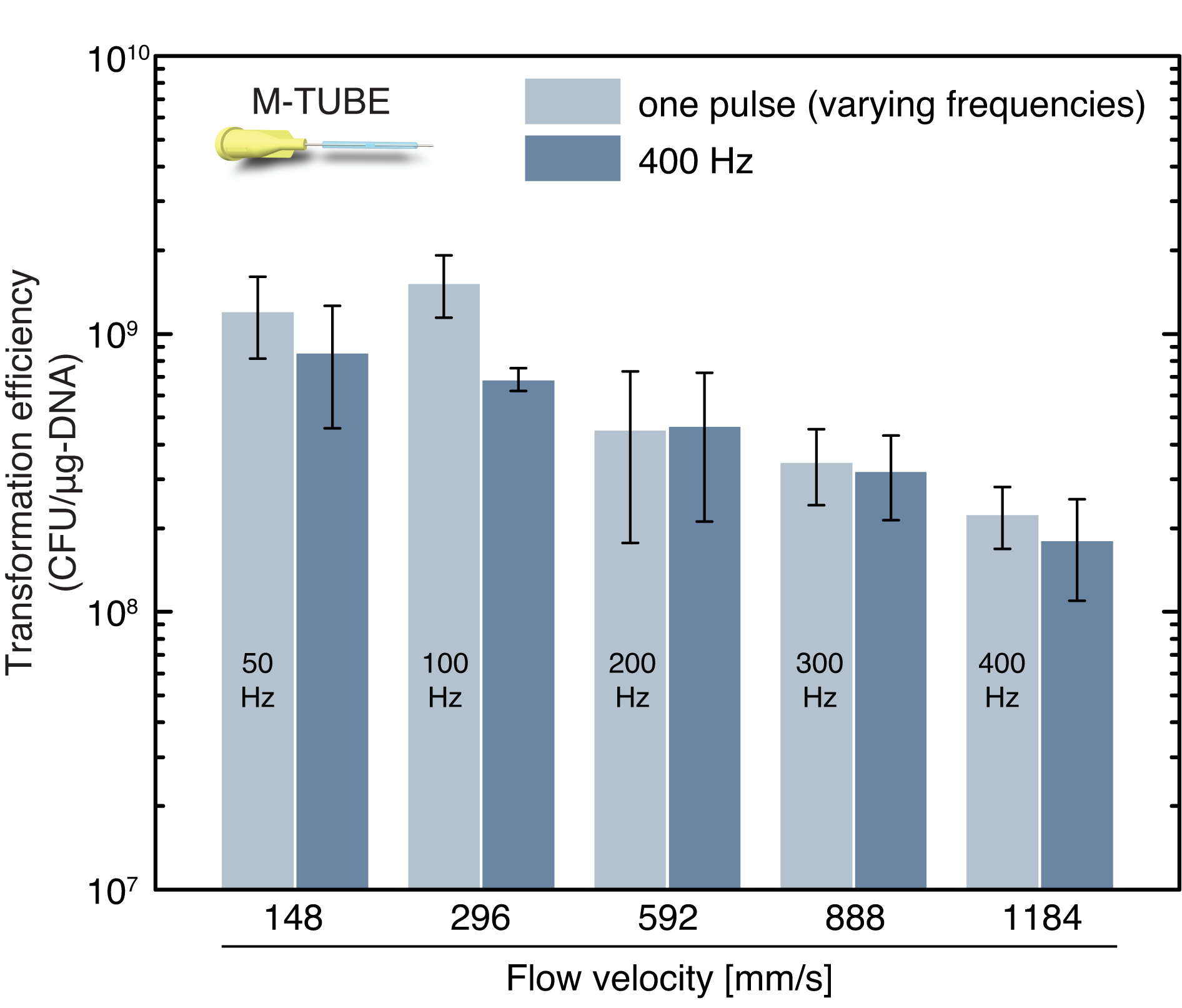

Supplement: S2 Fig — With M-TUBE devices, electroporation efficiency was largely independent of the applied AC field frequency. For M-TUBE devices, a voltage of ±2.50 kV (AC field) was applied, which results in an electric field of 8.33 kV/cm. Data represent the average (n ≥ 3) and error bars represent 1 standard deviation. The data underlying this figure can be found in S2 Data. AC, alternating current; M-TUBE, microfluidic tubing-based bacterial electroporation. (TIF) [file pbio.3001727.s010.tif]

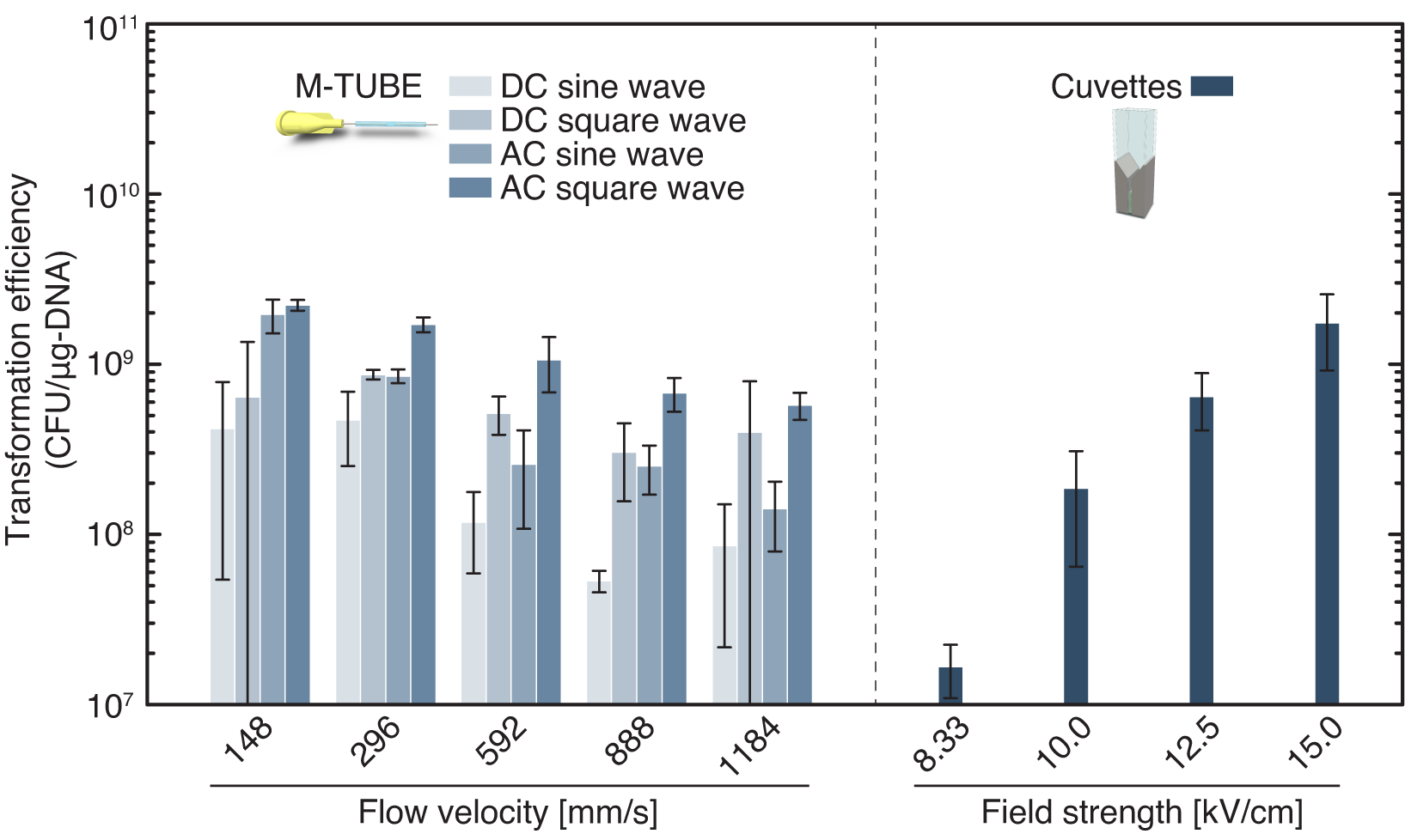

Supplement: S3 Fig — Using DC fields, M-TUBE devices achieved higher transformation efficiency than cuvettes using the same field strength or comparable efficiency using a lower field strength. Overall, electroporation efficiency and reproducibility were lower using DC fields compared with AC fields. For M-TUBE devices, a voltage of ±2.50 kV (AC field) or 2.50 kV (DC field with a duty cycle of 95%) was applied, which results in an electric field of 8.33 kV/cm. Data represent the average (n ≥ 3) and error bars represent 1 standard deviation. The data underlying this figure can be found in S2 Data. AC, alternating current; DC, direct current; M-TUBE, microfluidic tubing-based bacterial electroporation. (TIF) [file pbio.3001727.s011.tif]

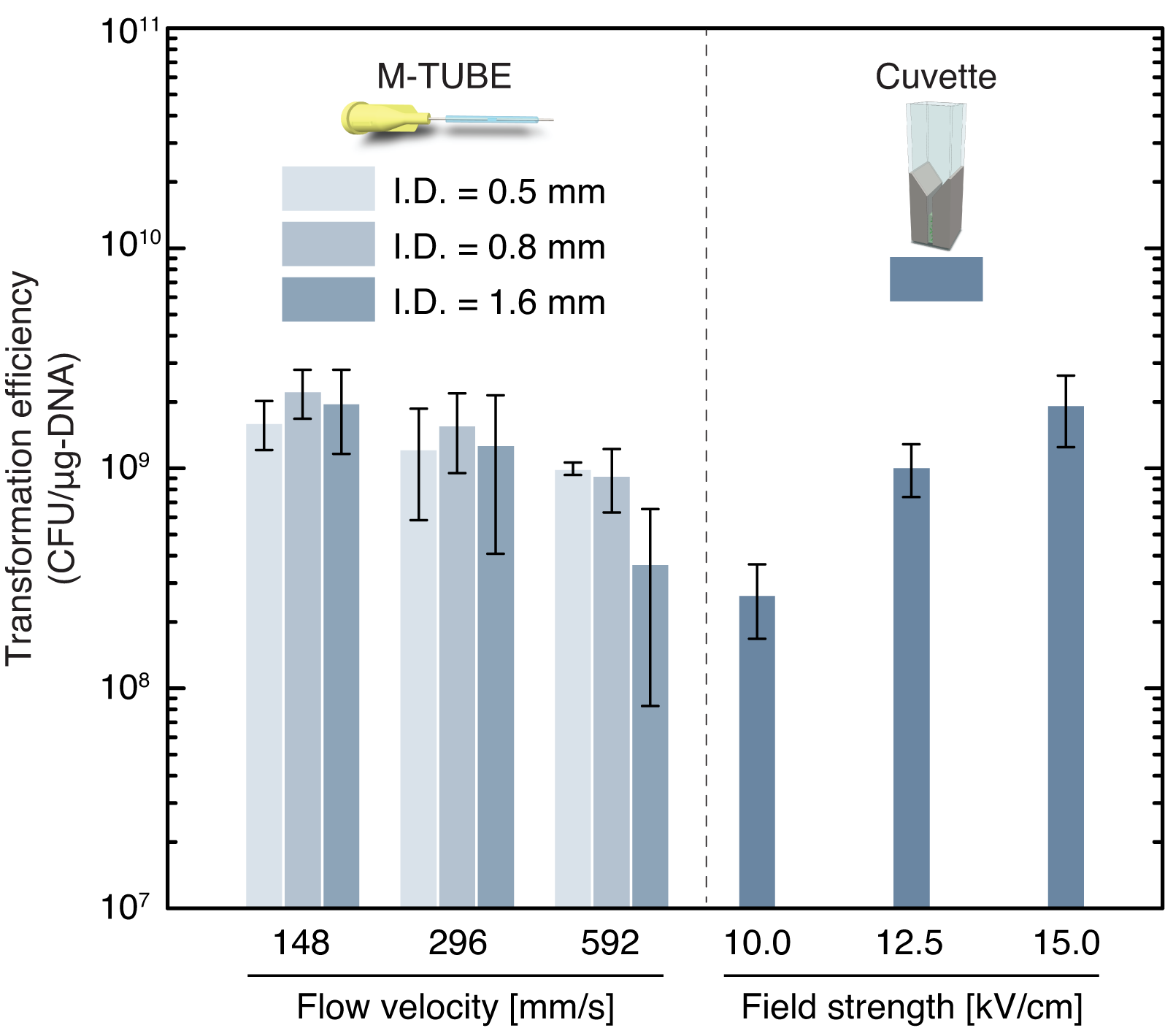

Supplement: S4 Fig — To further evaluate the scalability of M-TUBE, M-TUBE devices made using plastic tubing with 0.5-mm, 0.8-mm, and 1.6-mm inner diameters and compared to conventional cuvettes. A voltage of ±2.50 kV (AC field) was applied to each M-TUBE device, resulting in an electric field of 8.33 kV/cm. The same batch of cells was used to conduct electroporation with 0.2-mm cuvettes and various voltages as a comparison. Data represent the average (n ≥ 3) and error bars represent 1 standard deviation. The data underlying this figure can be found in S2 Data. AC, alternating current; M-TUBE, microfluidic tubing-based bacterial electroporation. (TIF) [file pbio.3001727.s012.tif]

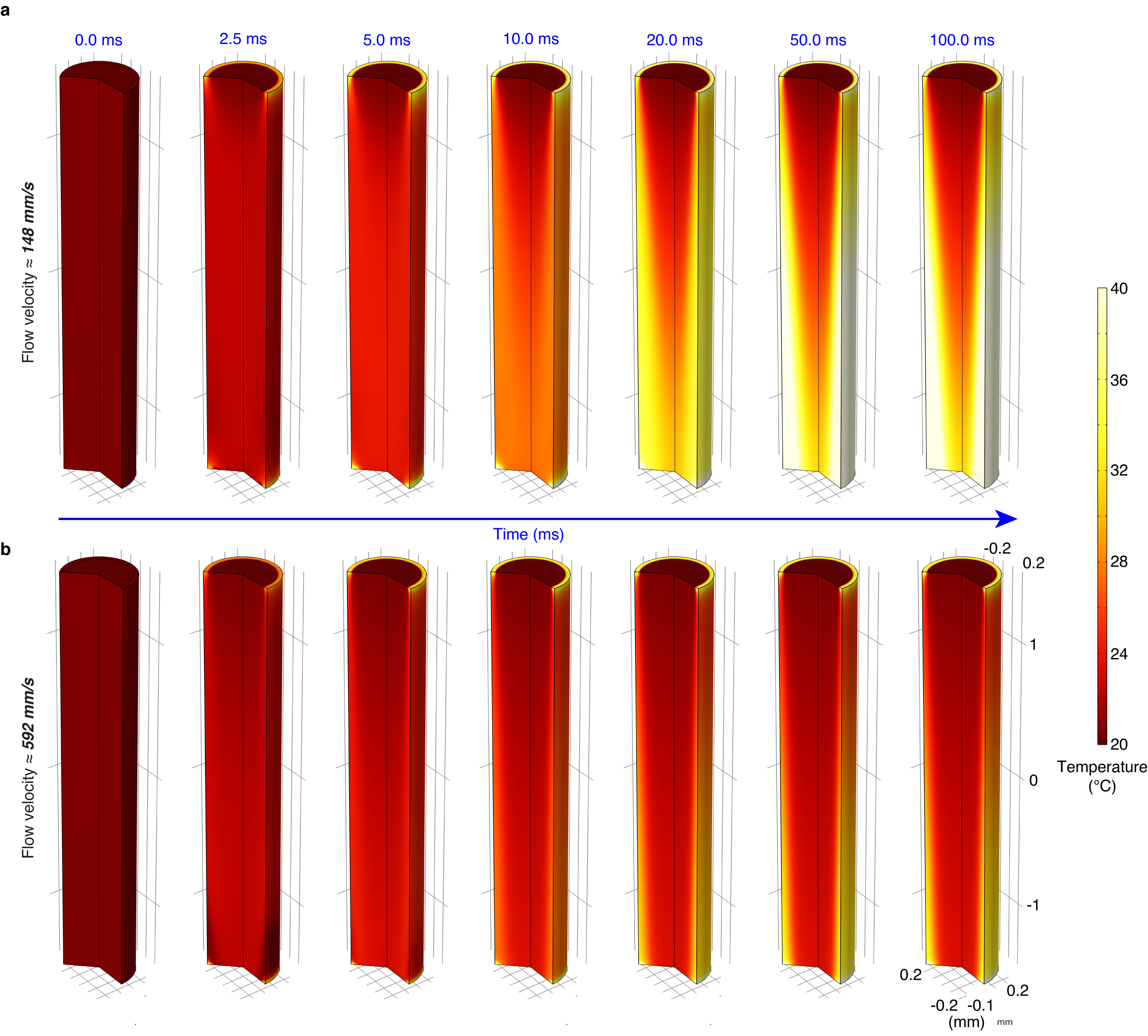

Supplement: S5 Fig — Cell samples were introduced into the microchannel at a fluid velocity of (a) 148 mm/s or (b) 592 mm/s. Simulations predicted higher and more uneven temperature increases for lower fluid velocity. The M-TUBE geometry used for simulations was 500 μm in diameter and 3 mm in length, and a voltage of 2.50 kV was applied, which leads to an electric field of 8.33 kV/cm. The initial temperature of the cell sample was 20°C. M-TUBE, microfluidic tubing-based bacterial electroporation. (TIF) [file pbio.3001727.s013.tif]

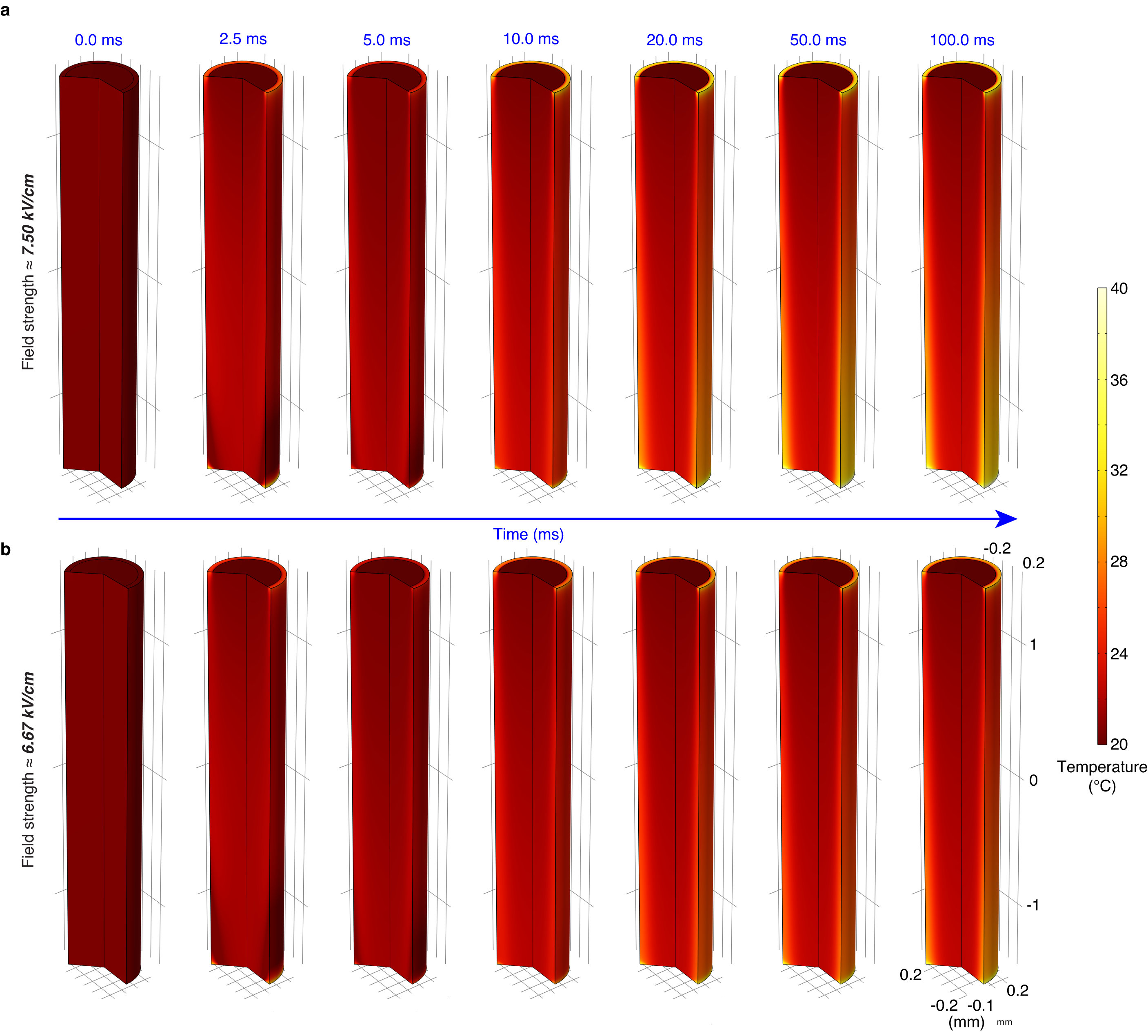

Supplement: S6 Fig — Voltages of (a) 2.25 kV (7.50 kV/cm) or (b) 2.00 kV (6.67 kV/cm) were applied. The M-TUBE geometry used for simulations was 500 μm in diameter and 3 mm in length. Cell samples were flowed through the microchannel at a fluid velocity of 592 mm/s for both simulations. The initial temperature of the cell sample was 20°C. M-TUBE, microfluidic tubing-based bacterial electroporation. (TIF) [file pbio.3001727.s014.tif]

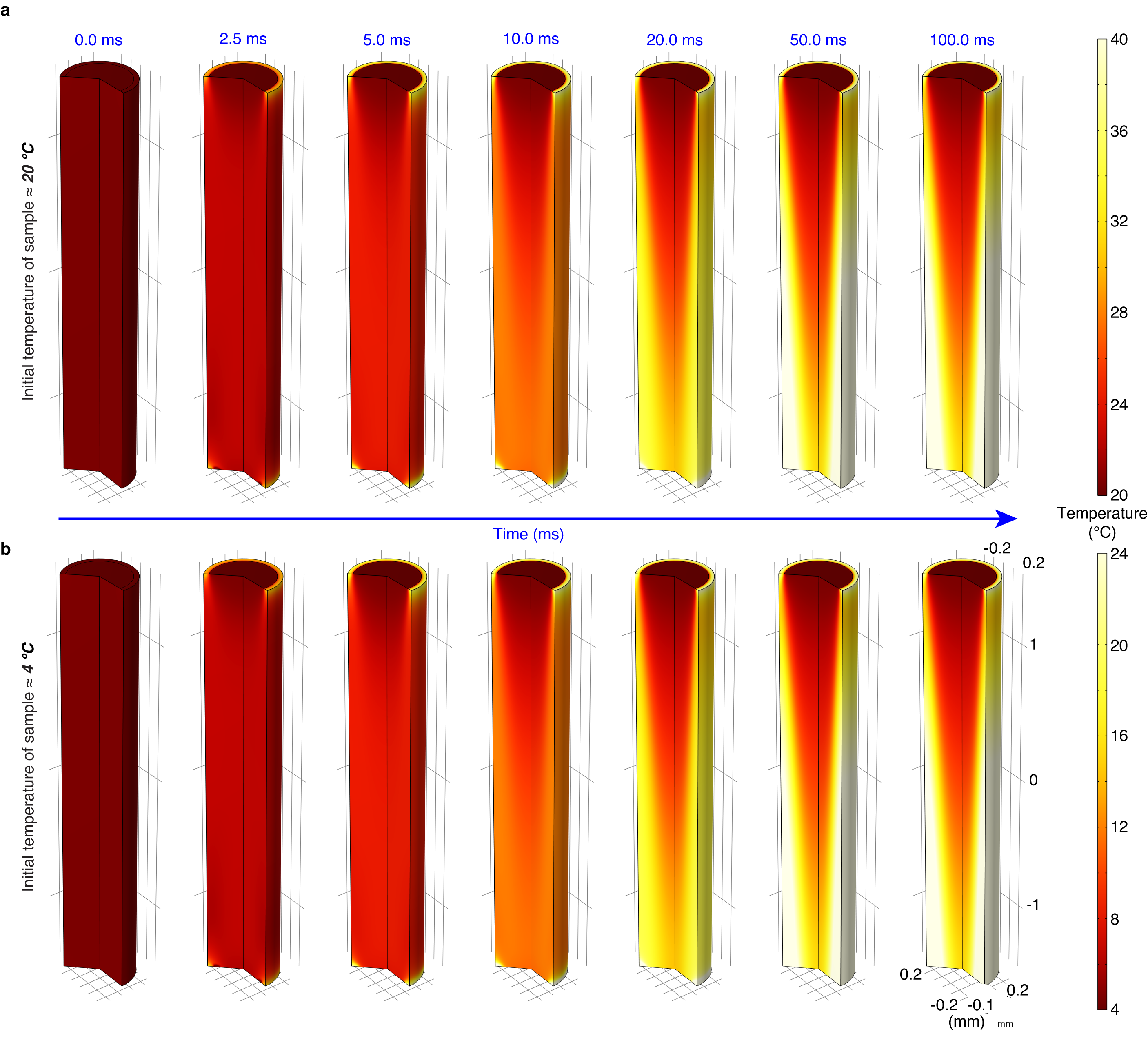

Supplement: S7 Fig — The cell sample was initialized with a temperature of (a) 20°C or (b) 4°C before flowing through the channel. The M-TUBE geometry used for simulations was 500 μm in diameter and 3 mm in length, and a voltage of 2.50 kV was applied, which leads to an electric field of 8.33 kV/cm. Cell samples flowed through the microchannel at a fluid velocity of 148 mm/s for both simulations. M-TUBE, microfluidic tubing-based bacterial electroporation. (TIF) [file pbio.3001727.s015.tif]

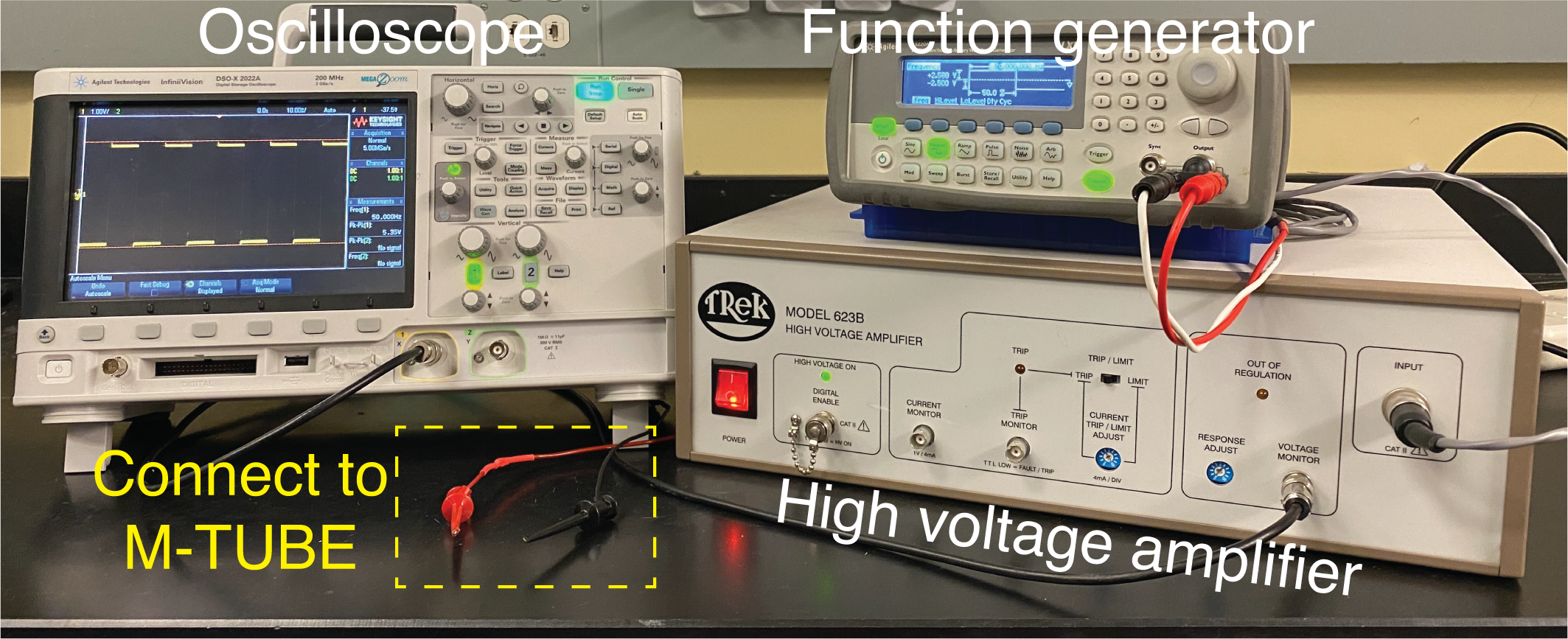

Supplement: S8 Fig — The system is composed of a function generator that allows for waveform programming, a high-voltage amplifier applied to the signal from the function generator, and an oscilloscope that allows for real-time monitoring of the amplified signal. (TIF) [file pbio.3001727.s016.tif]

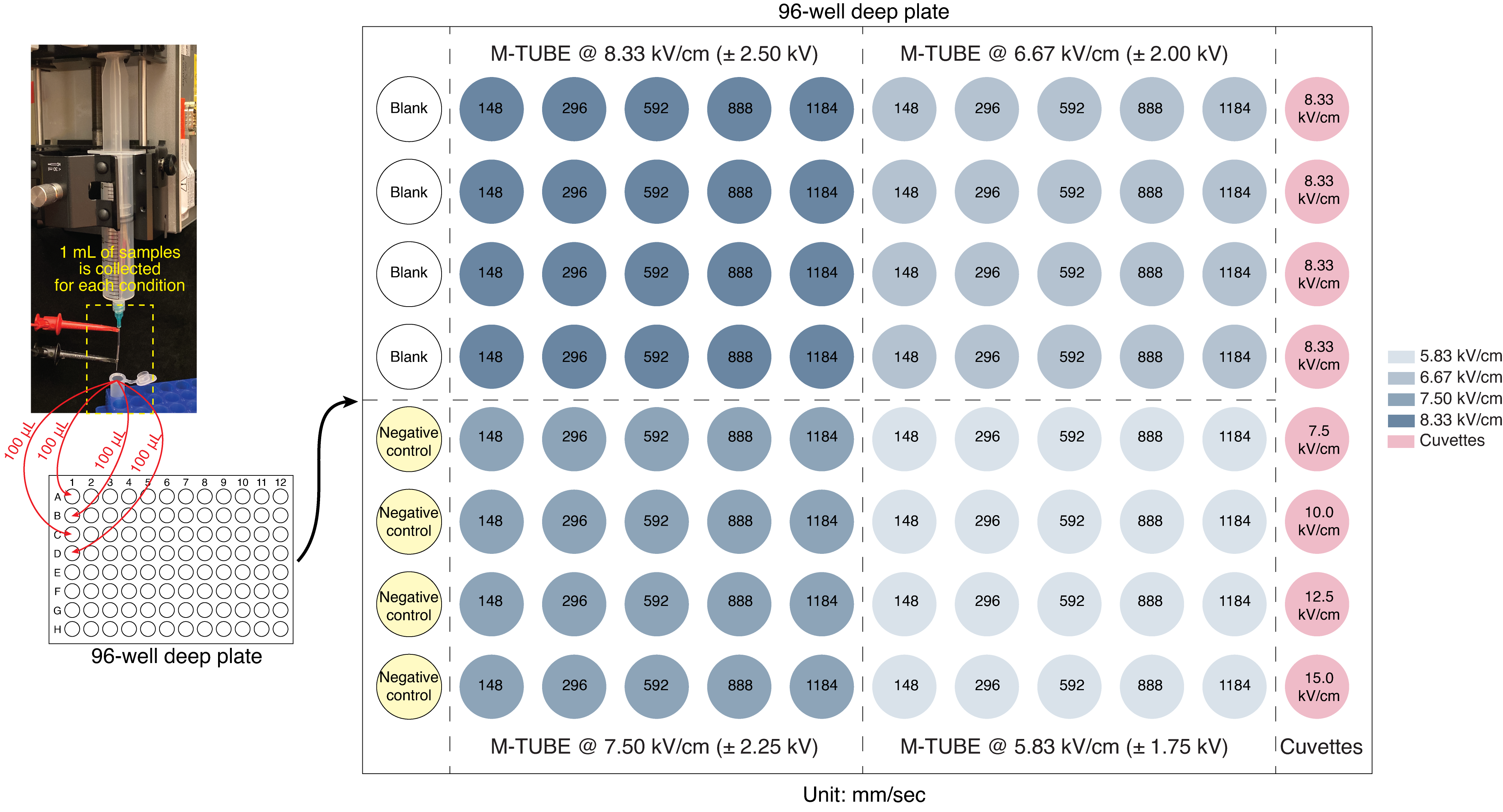

Supplement: S9 Fig — One milliliter of electroporated cells was collected for each combination of electroporation conditions tested. One hundred microliters were dispensed from each 1-mL sample into each of 4 designated wells containing 900 μL of LB recovery medium. For cuvette experiments, all of the volume aspirated from each cuvette was dispensed into a well. LB, Luria broth. (TIF) [file pbio.3001727.s017.tif]

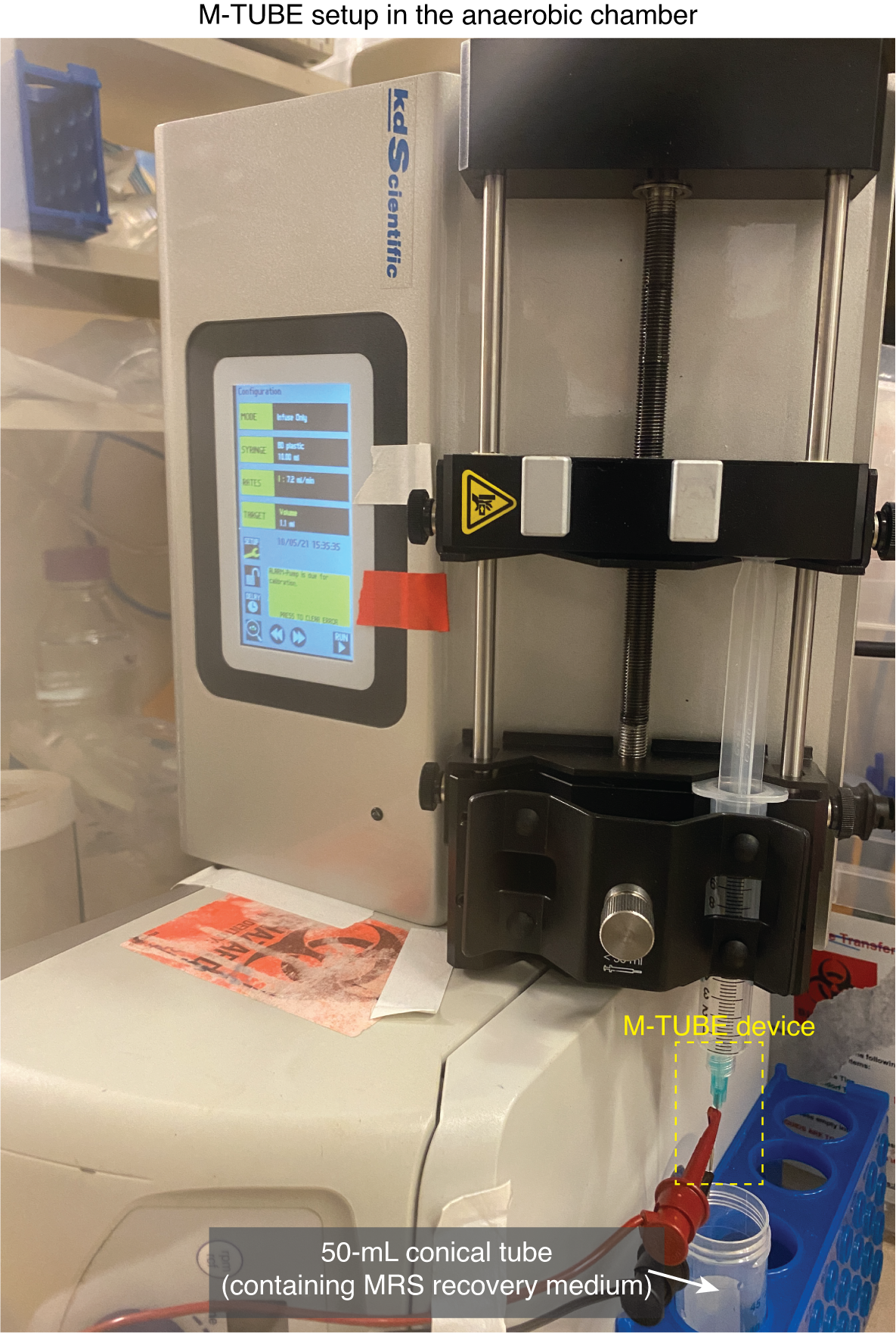

Supplement: S10 Fig — The M-TUBE device can be easily and conveniently set up in an anaerobic chamber. The photograph also shows that placing a collection tube (reservoir) directly underneath the fluid as it exits the M-TUBE device would enable the direct and automated transfer of electroporated cells into recovery medium. M-TUBE, microfluidic tubing-based bacterial electroporation. (TIF) [file pbio.3001727.s018.tif]

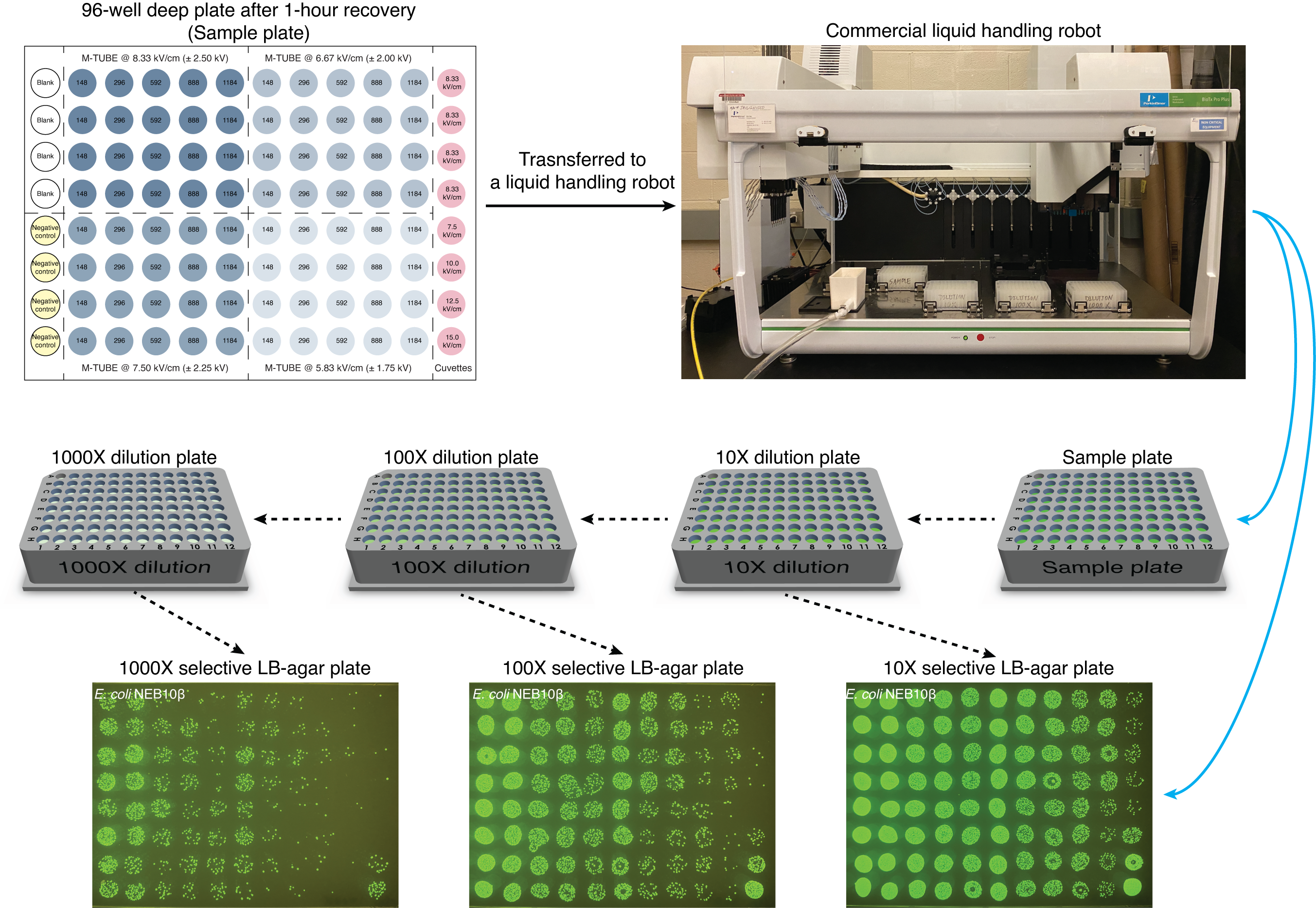

Supplement: S11 Fig — After 1 h of recovery, the 96-well deep plate that contains electroporated samples was mounted on a liquid-handling robot. By leveraging the capabilities of the robot, we used the M-TUBE device to test a wide range of electroporation conditions, each with at least 3–4 technical replicates, while removing the need for extensive manual pipetting for sample transfer, sample dilution, and sample plating. Strain shown is E. coli NEB10β. M-TUBE, microfluidic tubing-based bacterial electroporation. (TIF) [file pbio.3001727.s019.tif]
